# Mammalian Alpha Arrestins Link Activated Seven Transmembrane Receptors to Nedd4 Family E3 Ubiquitin Ligases and Interact with Beta Arrestins

**DOI:** 10.1371/journal.pone.0050557

**Published:** 2012-12-07

**Authors:** Fortune F. Shea, Jennie L. Rowell, Yechaowei Li, Tien-Hsien Chang, Carlos E. Alvarez

**Affiliations:** 1 Center for Molecular and Human Genetics, The Research Institute at Nationwide Children's Hospital, Columbus, Ohio, United States of America; 2 College of Nursing, The Ohio State University, Columbus, Ohio, United States of America; 3 Genomics Research Center, Academia Sinica, Taipei, Taiwan; 4 College of Medicine, The Ohio State University, Columbus, Ohio, United States of America; 5 College of Veterinary Medicine, The Ohio State University, Columbus, Ohio, United States of America; Yale Medical School, United States of America

## Abstract

The complement of fungal cell surface proteins is widely regulated by ubiquitination of membrane proteins, which results in their endocytosis and vacuolar degradation. For diverse fungal transporters, the specificity of ubiquitination is conferred by alpha arrestin adaptors, which recruit the Nedd4 family E3 ubiquitin ligase Rsp5. A recent study showed that one mammalian alpha arrestin also mediates ubiquitination and lysosomal trafficking of an activated plasma membrane receptor. Here we first screen all five widely-expressed human alpha arrestins for subcellular localization in ligand-stimulated and -unstimulated cells overexpressing the seven transmembrane receptor vasopressin 2. We then characterize the effects of alpha arrestins ARRDC3 and ARRDC4 upon activation of the seven transmembrane receptors vasopressin 2 and beta adrenergic 2. Using biochemical and imaging approaches, we show that ligand-activated receptors interact with alpha arrestins, and this results in recruitment of Nedd4 family E3 ubiquitin ligases and receptor ubiquitination – which are known to result in lysosomal trafficking. Our time course studies show these effects occur in the first 1–5 minutes after ligand activation, the same time that beta arrestins are known to have roles in receptor endocytic trafficking and kinase signaling. We tested the possibility that alpha and beta arrestins function coordinately and found co-immunoprecipitation and colocalization evidence to support this. Others recently reported that Arrdc3 knockout mice are lean and resistant to obesity. In the course of breeding our own Arrdc3-deficient mice, we observed two novel phenotypes in homozygotes: skin abnormalities, and embryonic lethality on normal chow diet, but not on high fat diet. Our findings suggest that alpha and beta arrestins function coordinately to maintain the optimal complement and function of cell surface proteins according to cellular physiological context and external signals. We discuss the implications of the alpha arrestin functions in fungi having evolved into coordinated alpha/beta arrestin functions in animals.

## Introduction

Cellular homeostasis is regulated through a multitude of plasma membrane proteins that sense the environment. Cells must maintain or rapidly alter the proper complement of membrane proteins according to extracellular signals. Additionally, signaling of cell surface receptors can be turned off by distinct desensitization mechanisms, such as internalization and degradation. In the case of activated seven transmembrane receptors (7TMRs), desensitization can be initiated by receptor phosphorylation and beta arrestin (bArr)-binding-mediated dissociation of G protein signaling. However, despite many recent studies of internalization, little is known about the mechanisms responsible for endocytic trafficking to lysosomes. [1] Activated membrane receptors often have accelerated turnover and degradation. And this is frequently associated with receptor phosphorylation, ubiquitination, and endocytic trafficking. [1] For example, lysosomal trafficking of cell surface EGFR requires ubiquitination by the RING finger E3 ubiquitin ligase Cbl. Cell surface stability of the epithelial Na+ channel (ENaC) is also regulated by ubiquitination. ENaC is comprised of three subunits, each with a C-terminal PPxY (or PY) motif. Those PY motifs interact with the WW domains of Nedd4 family E3 ubiquitin ligases. The resulting ubiquitination of the channel results in its down regulation. [2] At least 15 other transporters, plasma membrane ATPases, and channels are known to be similarly regulated by ubiquitination. [3] Curiously, in six of the seven cases where the enzyme responsible was identified, it was a member of the WW-containing Nedd4 E3 ubiquitin ligase family. However, some of those membrane proteins lack PY motifs and presumably require adaptor proteins to mediate the interaction.

In 1998, Hicke and colleagues showed that internalization of the *S. cerevisiae* 7TMR Ste2 was dependent on ligand-induced hyperphosphorylation of its cytoplasmic tail. [4] They subsequently showed that mutagenesis of WW domains in the (Nedd4 E3) Rsp5 inhibits both ubiquitination and internalization/degradation of activated Ste2. [5] However, Ste2 does not have a PY motif, and it remains unknown how activated Ste2 recruits Rsp5. Regulated ubiquitination and endocytosis has been studied for a few mammalian 7TMRs: most notably beta 2 adrenergic (b2AR) and chemokine receptor CXCR4, but also others such as delta opioid, vasopressin V2R, and platelet activating factor. [6], [7], [8], [9], [10] Shenoy, Lefkowitz and colleagues reported that activated b2AR is quickly ubiquitinated, and that this modification is not necessary for internalization, but rather for trafficking to lysosomes and degradation. [6], [11] They showed that b2AR ubiquitination is dependent on the expression of bArr ARRB2 and on its ubiquitination by the RING family E3 ubiquitin ligase MDM2. And they also reported that ligand activated b2AR ubiquitination is mediated by recruitment of Nedd4 by the bArr ARRB2 (others attribute this to the alpha arrestin ARRDC3; see below and [12]). [11] Marchese and associates showed activated CXCR4 is ubiquitinated by ITCH/AIP4, a member of the Nedd4 E3 family, and that this is not required for internalization, but it is for lysosomal targeting. [13] More recently, the same group proposed that the bArr ARRB1 recruits ITCH to activated CXCR4. [14] Notably, bArrs do not have PY motifs, which are known to bind WW domains.

We and others recently noted the existence of previously unrecognized arrestin family proteins. [15], [16], [17], [18], [19], [20] We conducted phylogenetic studies and determined that the arrestin clan is ancient, with one family extant in bacteria and archaea – Spo0M. [15] In essentially all eukaryotes, the clan is made up of two families – Vps26 and arrestin (plants only have Vps26). [15] Most arrestins belong to the ancient/ancestral alpha arrestin (aArr) subfamily. The closely related beta and visual arrestins (b/vArrs) emerged relatively recently (in animals), beginning with duplication of an aArr. Mammals have six aArrs named Arrestin Domain Containing 1 through 5 (*ARRDC1–5*) and Thioredoxin Interacting Protein or Vitamin D Upregulated Protein 1 (*TXNIP* or *VDUP1*). The two mammalian bArrs are Beta arrestin 1 and 2 (*ARRB1/2*) and the two vArrs are S Antigen (*SAG*) and Arrestin 3 (*ARR3*). Mammals also have at least three Vps26 family genes. [15] bArrs have preserved the protein architecture of the ancestral Vps26/aArr – two homologous arrestin domains (Arr-N and C) separated by a small hinge and followed by a C-terminal Tail domain with little secondary structure. [15] The functional motifs identified in bArr tails are not recognizable in aArr tails, but fungi and animal aArrs (but not bArrs) have highly conserved PY motifs in their Tail domains (Fig. S1). [15] We computationally identified ∼20 proteins in the human genome that are likely to bind aArr PY motifs and nine of those are Nedd4 family E3 ubiquitin ligases. Before yeast Rod1 and Rog3 were known to be arrestins, it was shown that their PY motifs interact with the WW domains of the Nedd4 E3 Rsp5. [21] We thus proposed that one role of fungal/metazoan aArrs is to recruit WW proteins – including Nedd4 E3s – to activated cell surface proteins. [15] We also speculated that metazoan aArrs and bArrs are likely to heteroassociate as bArrs do. [15], [22]

Recently, the first signaling study of an animal aArr was published. [12] Nabhan, Pan and Lu showed that human ARRDC3 is required for ubiquitination and lysosomal degradation of activated b2AR. They determined that ARRDC3 PY motifs recruit Nedd4 to liganded b2AR, and showed that ARRDC3 siRNA or PY mutants blocked receptor ubiquitination and degradation. As part of a larger study, others replicated similar effects of b2AR/ARRDC3. [23] Others have also demonstrated in cell culture that mammalian aArrs interact with Nedd4 family E3s and with ubiquitin and components of the endosomal sorting complex required for transport (ESCRT) ALIX and Tsg101. [24] In a xenograft model, ARRDC3 was shown to repress human breast cancer tumorigenicity in mice, and mechanistic studies showed that it binds to, and induces internalization, ubiquitination and degradation of phosphorylated beta-4 integrin. [25] There are only significant numbers of published physiological studies of one aArr – TXNIP. [26] Generally more recent studies have begun to dissect the biological roles of ARRDC3 and ARRDC4. [17], [23], [26], [27], [28], [29] All three of these aArrs are involved in metabolic regulation, a feature conserved in yeast aArrs. [30], [31], [32], [33] All three also have tumor suppressor-like properties. [25], [26], [27], [28], [34], [35]

This is only the second investigation focused on 7TMR signaling roles of mammalian aArrs. We used cell culture models to study ARRDC3 and ARRDC4 in the context of ligand activation of two receptors who's endocytic trafficking is well characterized, b2AR and Vasopressin 2 receptor (V2R). [36] Notably, 7TMRs can be classified according to whether they have transient (e.g., b2AR) or sustained (e.g., V2R) interactions with bArrs, which determines whether an activated receptor will be recycled or trafficked to lysosomes, respectively; and this is, in part, regulated by ubiquitination of bArrs. [37] Here we report evidence supporting our previous proposal that aArrs 1) are involved in endocytic trafficking, 2) recruit WW proteins, including Nedd4 family E3s, to activated receptors and 3) heteroassociate with bArrs. [15]

## Results

### Subcellular localization of alpha arrestins in receptor-stimulated and -unstimulated cells

**Figure 1:**
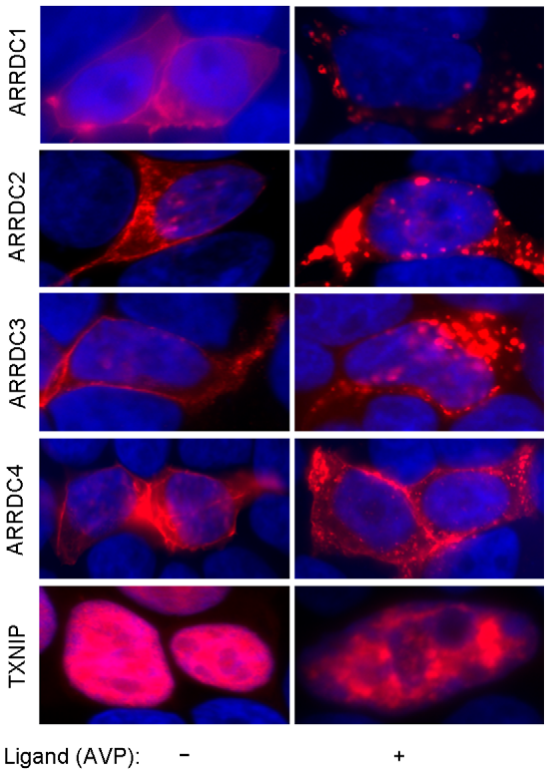
Subcellular localization of alpha arrestins. Vasopressin receptor 2 HA-V2R-V5-stably transfected HEK293T cells were transiently transfected with expression constructs of alpha arrestins with C-terminal fusions of fluorescent protein mCherry. Unstimulated or 1 uM arginine vasopressin (AVP)-stimulated (15 m) cells were fixed, and epifluorescence images were captured using an AxioVision confocal microscope.

Five of six mammalian aArrs, but not ARRDC5 (which has overall divergent protein sequence, no PY motifs, and testis-predominant expression), are broadly expressed, suggesting they may be promiscuous like bArrs. [15] To ascertain subcellular expression patterns, we used confocal imaging of the human proteins in transfected human cells. Figure 1, shows V2R-stably transfected HEK293T (293T-V2R) transiently transfected with aArr C-terminally fused with fluorescent protein mCherry. Artifactual localization of fluorescent protein tags is ruled out by a pattern of diffuse cytoplasmic expression of fluorescent protein alone (not shown) and by the different localization patterns of ARRDC1, ARRDC2/3/4, TXNIP and ARRB2. ARRDC2, 3 and 4 have similar constitutive subcellular localization at the plasma membrane and in cytoplasmic vesicles. ARRDC1 had more variable constitutive subcellular localization that could be similar to ARRDC2/3/4 or have more diffuse/less punctate cytoplasmic expression. At 15 m post 1 uM AVP stimulation of V2R, all five aArrs showed altered subcellular localization. ARRDC1/2/3/4-mCherry in AVP-activated 293T-V2R cells were predominantly localized to cytoplasmic vesicles. A minute proportion of the fluorescence was seen as nuclear puncta, as is seen most prominently here for ARRDC2/3 (Fig. 1); further studies are necessary to rule out the possibility those are cytoplasmic invaginations into the nucleus. TXNIP-mCherry remained nuclear, but frequently transformed from a diffuse to a punctate pattern.

### Ligand activated receptor-alpha arrestin interactions and trafficking

**Figure 2:**
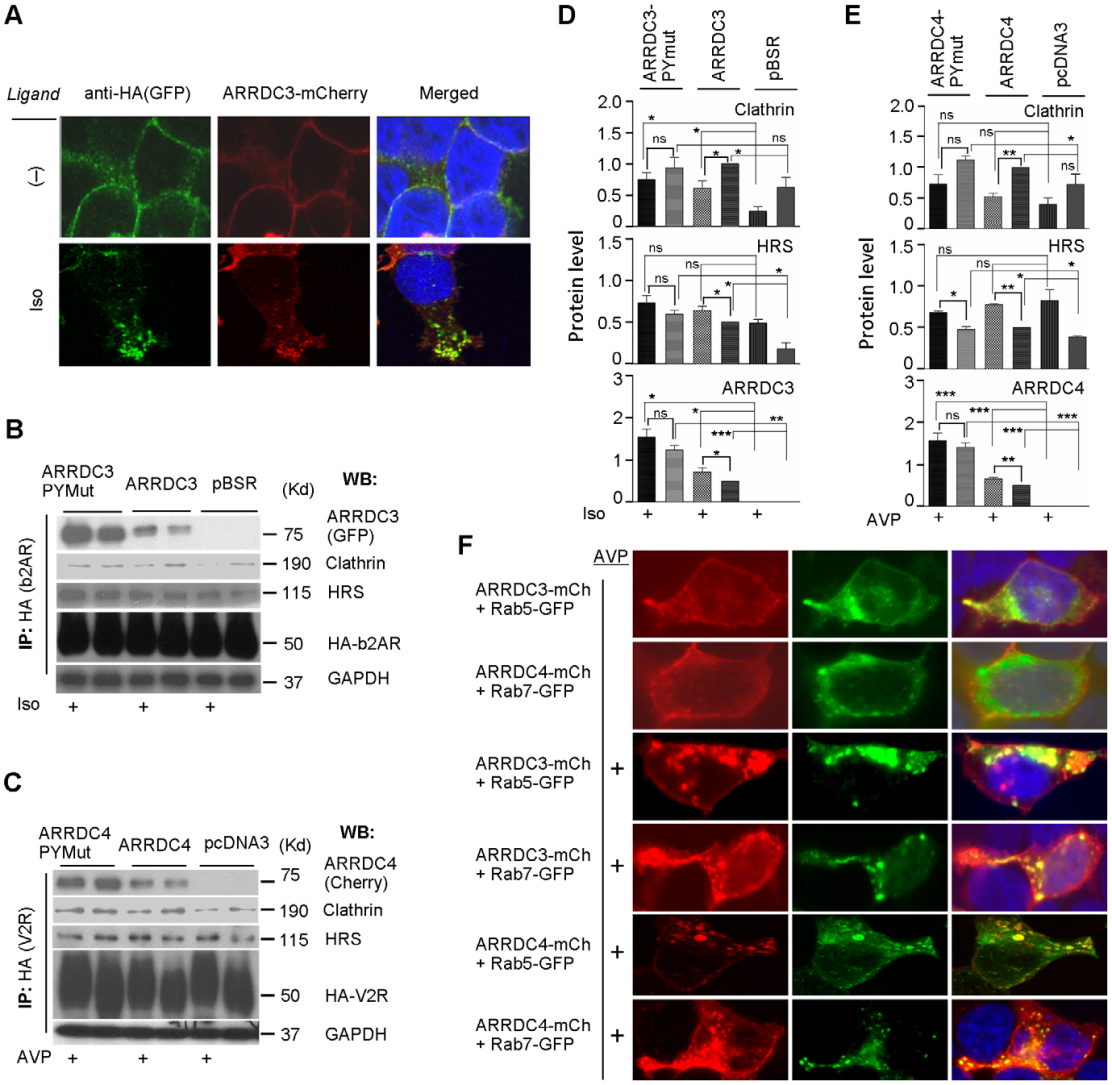
Subcellular colocalization and coIP of alpha arrestins with activated GPCR, clathrin, and endosome markers. (*A*) Subcellular colocalization of alpha-arrestin-mCherry in receptor HA-b2AR permanent cell lines. HEK-293T cells stably expressing HA-b2AR were transiently cotransfected with ARRDC3-mCherry construct. 24 h after transfection, cells were serum-starved for 2 h, treated or not with 1 uM isoproterenol (Iso) ligand for 30 m, and then washed, and fixed and permeabilized. HA-b2AR was stained with rabbit anti-HA antibodies followed by incubation with Alexa Fluor 488-conjugated goat anti-rabbit secondary antibody. Epifluorescence images were captured using AxioVision or Zeiss 510 META confocal microscope (receptor, *green*; aArr, *red*). (*B*) Co-immunoprecipitation of aArr ARRDC3 with b2AR, clathrin and Hrs. HEK-293T cells were transiently cotransfected with HA-b2AR-V5 plus either empty vector, pBSR-ARRDC3-GFP, or pBSR-ARRDC3 PY motif mutant construct respectively. After 24 h incubation, cells were serum-starved for 2 h and treated or not with 1 uM Iso for 30 m. The cells were lysed, and lysates were immunoprecipitated (IP) and analyzed by western blot (WB). (*C*) Co-immunoprecipitation of aArr ARRDC4 with V2R, clathrin, and Hrs. HEK-293T cells were transiently cotransfected with HA-V2R-V5 plus either empty vector, pcDNA3-ARRDC4-Flag, or pcDNA3-ARRDC4 PY motif mutant construct respectively. After 24 h incubation, cells were serum-starved for 2 h and treated or not with 1 uM AVP for 30 m. The cells were lysed, the lysates were immunoprecipitated (IP), and analyzed as above. (*D, E*) Histograms of B and C from three independent experiments including the mean (+/− S.D.) and p-values calculated by paired, two-tailed t-tests on signal from untreated and ligand-treated samples versus their respective vector control are denoted by thin lines and treated vs. untreated samples are denoted by bold lines (***, p<0.001; **, p<0.01; *p<0.05). (*F*) Subcellular colocalization of alpha-arrestin-mCherry with early (Rab5) and late (Rab7) endosomal markers in HA-V2R-V5 permanent cells. HA-V2R-V5 permanent cells were transiently co-transfected with ARRDC3- or ARRDC4-mCherry construct with Rab5- or Rab7-GFP construct. After 24 h, transfected cells were serum-starved, treated or not with 1 uM AVP for 30 m, and fixed with 4% paraformaldehyde. Epifluorescence images were captured using AxioVision confocal microscope (aArr, *red*; Rab, *green*).

We next considered whether the ligand-induced effects above are indicative of aArr-receptor interaction that could be linked to co-endocytic-trafficking. We chose to focus on ARRDC3 and 4 because of their plasma membrane subcellular localization patterns. We transiently transfected stable lines 293T-b2AR and 293T-V2R (which have N-terminal hemagglutinin, HA, affinity tags on each receptor) with ARRDC3- and ARRDC4-mCherry, respectively. [See end of Introduction for rationale of receptor choice. The matching of receptor and aArr was based on reduced ARRDC4/b2AR co-immunoprecipitation (coIP) effects, which have not been thoroughly characterized (not shown), but ARRDC3 shows robust colocalization (Fig. 1) and coIP (not shown) with activated V2R.] When we treated those cells for 30 m with their agonist ligands isoproterenol (1 uM Iso) and arginine vasopressin (1 uM AVP), respectively, we saw that, in both cases, there was strong endocytic trafficking of HA-tagged receptors and colocalization with aArr-mCherry (Fig. 2A and data not shown). Similar results were observed conducting the same experiments in HeLa cells co-transfected with the same constructs (not shown).

We next conducted coIP studies using 293T cells transiently co-transfected with HA- tagged b2AR or V2R, respectively, plus wild type (WT), or PY mutant (PYmut; see “*Alpha arrestin PY motifs…*” below) alpha arrestin C-terminally tagged with fluorescent protein (GFP or mCherry). As HA-receptors have similar electrophoretic mobility to immunoglobulin heavy chains, we first established that HA-receptors can be detected by coIP Western blotting without cross-reaction with the IP antibody (Fig. S2A/B; see three lanes without HA-receptor). IP of HA (receptor) and western blotting of either ARRDC3 or 4 revealed that both b2AR/ARRDC3-WT and V2R/ARRDC4-WT significantly increased coIP after 30 m of ligand treatment (Fig. 2B–E; see also S2B). Importantly, coIP was robust in the absence of chemically-induced protein cross-linking; this suggests the interaction is of a moderate to high affinity. Notably, the protein levels of aArr-PYmut are always very significantly higher than WT. That effect is specific to the overexpressed alpha arrestin and not to co-transfected receptors (Figs. 2B/C and Fig. S2C). As suggested by a reviewer of this manuscript, we tested for evidence of proteosomal or lysosomal degradation as the mechanism responsible. Proteosomal inhibition (10 uM MG132) in HeLa cells co-transfected with ARRDC4 and V2R resulted in elevated levels of WT, but not PY mutant, ARRDC4 (Fig. S2C). However, those increased WT protein levels did not rise to those of PY mutant ARRDC4. Treatment with chloroquine (100 uM), which inhibits lysosomal function and autophagy, did not result in increased ARRDC4 levels (rather they decreased).

In the same experiments, we also measured coIP of two major proteins involved in early endocytic trafficking, clathrin and hepatocyte growth factor-regulated tyrosine kinase substrate (Hrs), a component of the endosomal sorting complexes required for transport (ESCRT)-0 complex. This 30 m post-stimulation snapshot showed slightly reduced clathrin and slightly increased Hrs coIP with receptor. aArr-PYmut showed the same trend as WT, but had a decreased difference between stimulated and unstimulated cells. To test whether the above cytoplasmic vesicles may be endosomes, we co-expressed fluorescent aArrs and early and late endosome markers Rab5 and Rab7, respectively, in 293T-V2R cells (Fig. 2F). Without ligand stimulation both Rabs were strongly expressed in vesicular structures. 30 m following ligand stimulation there was colocalization of aArrs in a subset of both Rab5- and Rab7-containing cytoplasmic vesicles. These results suggest that ligand-activated receptor/aArr are internalized and trafficked together from early to late endosomes, but this will require confirmation by co-detection of receptor/aArr/Rab marker proteins in stimulated cells.

### Alpha arrestins are involved in early 7TMR signaling events

**Figure 3:**
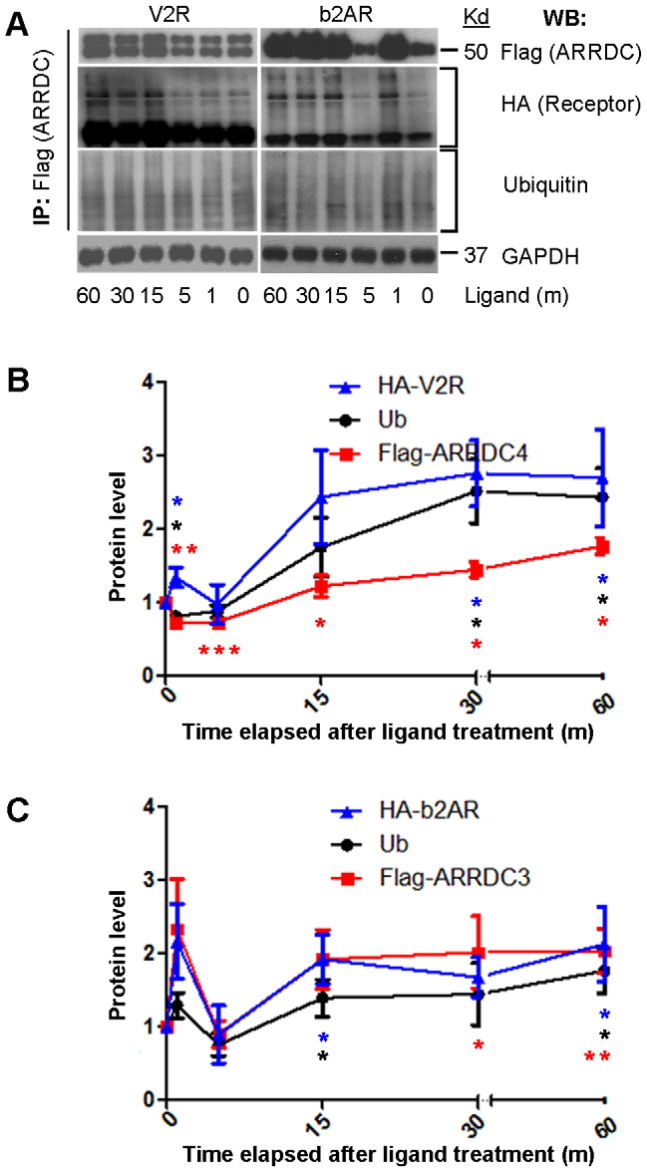
Time course analysis of ligand-stimulated aArrs. (*A*) HEK-293T cells were transiently cotransfected with HA-b2AR-V5 plus pBSR-ARRDC3-Flag, or HA-V2R-V5 plus pcDNA3-ARRDC4-Flag respectively. After 24 h incubation, cells were serum-starved and treated with 1 uM Iso or AVP for 1, 5, 15, 30, and 60 m. The cells were lysed, and lysates were immunoprecipitated (IP) and analyzed by western blot (WB). Note ARRDC4-Flag protein appears as two bands; we believe this is also the case for ARRDC4-mCherry (Fig. 2C), but that is less evident due to the large size of the fusion protein. The basis of this is not yet determined. (*B, C*) The histogram of A with mean (+/− S.D.) and p-values calculated by comparing levels of ligand-treated samples with respective vector controls (paired, two-tailed t-tests from three independent experiments; ***, p<0.001; **, p<0.01; *p<0.05).

While the studies above are focused on 30 m post-stimulus, we also conducted time course studies of liganded b2AR-ARRDC3 and V2R-ARRDC4 interactions. We conducted IP of aArr (Flag) and western blotting for receptor (HA), ubiquitin and aArr (Flag). [As Flag-alpha arrestins have similar electrophoretic mobility to immunoglobulin heavy chains, we first established that Flag-alpha arrestins can be detected after IP without cross-reaction with IP antibody (Fig. S2D).] This revealed a similar pattern for both receptor-aArr combinations: there was a rapid increase of aArr-receptor interaction observed at 1 m followed by a decrease at 5 m and then a more gradual increase that plateaus after 15–30 m (Fig. 3). For ARRDC3-b2AR, both ubiquitination and ARRDC3 levels are parallel to those of b2AR. However, for V2R, receptor coIP is increased while ARRDC4 and ubiquitin are decreased at 1 m (then they look similar to ARRDC3-b2AR from 5–60 m (Fig. 3)). Further studies are necessary to test the possibility that the peak for ligand dependent ARRDC4-V2R interaction and ubiquitination occurs before 1 m. In Discussion we describe how our aArr findings are similar to those for bArr in the context of b2AR and V2R activation.

### Alpha arrestin PY motifs bind WW domains in signaling associated proteins

At least in fungi and animals, most aArr tails (but not bArrs) contain multiple PY motifs. [15] With the exception of ARRDC5, all mammalian aArrs have a pair of PY motifs. Analysis of their Tail domains across hundreds of millions of years of evolution shows the highest levels of conservation are centered on these two PY motifs (Fig. S1). PY motifs are known to bind WW domains, SH3-like domains present in diverse signaling and cytoskeletal proteins. [38] Two of the PY motifs are conserved in distantly related aArrs TXNIP and ARRDC2/3/4, while those of ARRDC1 have different flanking consensus sequences and locations in the tail. [15] WW domains are classified by the motif each recognizes: PPXY/LPXY (“PY”), PPLP, P/G/M-rich, phospho-(S/T)P and P/R-rich. [38], [39] We used WW-domain specificities [38] to computationally identify human WW domain proteins likely to bind aArr PY motifs. That yielded ∼20 proteins in the human genome, including the nine Nedd4 family E3 ubiquitin ligases (not shown).

**Figure 4:**
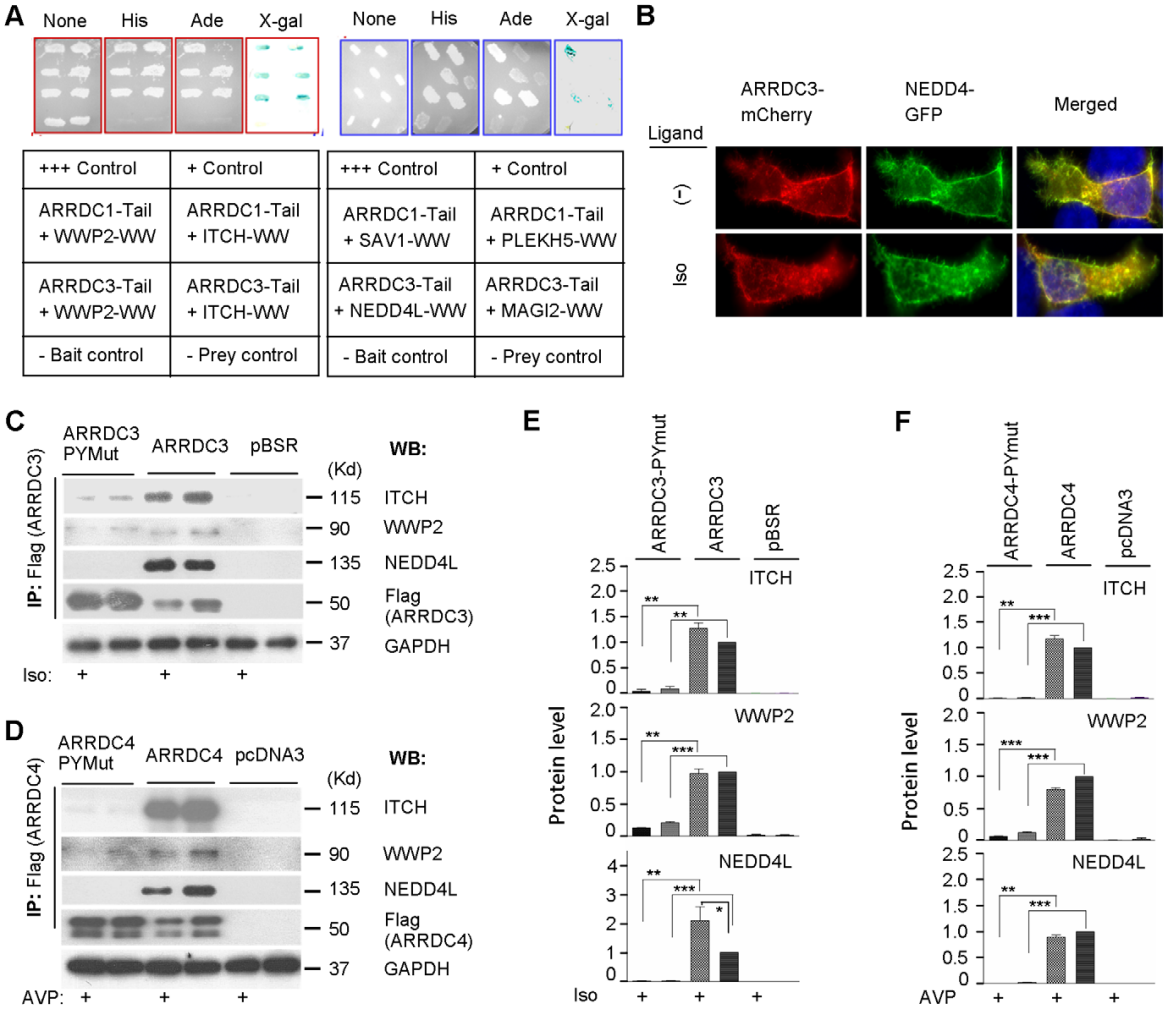
Recruitment and activation of Nedd4 E3 ligases by alpha arrestins. (*A*) Interactions of PY motif-containing alpha arrestin tails and WW region fragments demonstrated by yeast two-hybrid analysis. Bait plasmid (pDEST32-ARRDC1 or -ARRDC3) which contain two sets of PY motifs and prey plasmid (pDEST22-ITCH, -WWP2, -SAV1, -PLEKHA5, -Nedd4L, or -MAGI-2) which contain 1–4 sets of WW domains were transformed into competent yeast strain MaV203. pEXP32/Krev1 plus pEXP22/RalGDS-wt was included as strong positive control, pEXP32/Krev1 plus pEXP22/RALGDS-m1 weak positive control, pEXP32/Krev1 plus pEXP22/RALGDS-m2 and empty bait vector pDEST32 plus empty prey vector pDEST22 negative controls. Positive clones growing on SD/-Trp/-Leu/-His/-Ura plates are selected and the activity of lacZ reporter gene is examined with colony-lift filter assay by X-gal. (*B*) Subcellular colocalization of alpha-arrestin-mCherry and Nedd4 in HA-b2AR permanent cell lines. HA-b2AR permanent cells were transiently co-transfected with ARRDC3 -mCherry and pBJ-Nedd4-myc constructs. After 24 h, transfected cells were serum-starved, treated with 1 uM Iso for 30 m, and fixed. Nedd4 was stained with mouse monoclonal anti-myc antibody followed by incubation with Alexa Fluor 488-conjugated goat anti-mouse second antibody. Confocal epifluorescence images were obtained (aArr, *red*; Nedd4, *green*). (*C*) Co-immunoprecipitation of alpha arestin ARRDC3 and Nedd4 family E3 ubiquitin ligases with overexpressed b2AR. Empty vector, pBSR-ARRDC3-Flag, or pBSR-ARRDC3-Flag PY motif mutant construct was cotransfected respectively with pcDNA3-HA-b2AR-V5. After 24 h incubation, cells were serum-starved and treated or not with 1 uM Iso for 5 m. The transfected cells were lysed, lysates were immunoprecipitated (IP), and analyzed by western blot (WB). (*D*) Co-immunoprecipitation of aArr ARRDC4 and Nedd4 family E3 ubiquitin ligases with overexpressed V2R, unstimulated or 5 m post 1 uM AVP stimulation. (*E, F*) Histograms of C and D from three different experiments; p-values were calculated by comparing levels of ligand-treated or non-treated samples with their respective vector control (p value levels and thin/bold lines used as in Fig. 2).

We initiated PY binding studies by testing aArr Tail and WW domain fragments by yeast two hybrid analysis (Fig. 4A). The many published successes with PY-WW yeast two hybrid interactions show that neither PY motifs nor WW domains are generally prone to self-activating false positives. [40] There are two classes of aArr PY motifs defined by their positions, distance apart and conserved flanking sequence (ARRDC1 and TXNIP/ARRDC2/3/4). [15] We tested eight combinations of tail fragments (containing both PY motifs) from ARRDC1 and 3 with complete WW regions of select candidate partners. We found that mammalian aArr tails can interact with WW domain-containing protein fragments. Both the Nedd4 family E3s that we tested with both aArr tails, ITCH/AIP4 and WWP2, were positive. The ARRDC3 tail also bound both the E3 NEDD4L and MAGI2 (membrane-associated guanylate kinase inverted-2) WW domains. [MAGI proteins, which have six PDZ, two WW and a guanylate kinase-like domains, interact with 7TMRs and are involved in receptor internalization and protein scaffolding. [41]] Our results also show some specificity – i.e., the ARRDC1 tail strongly binds WW domains from two E3s, but not from SAV1 or PLEKHA5 (the latter were selected for their closely spaced WW domains, as ARRDC1 has closely spaced PY motifs). Comparisons to control interactions show that some are high affinity (e.g., both aArr tails binding to ITCH and WWP2). These findings show that aArr PY motifs can bind WW domains from different protein families.

### Alpha arrestins recruit Nedd4 family E3 ubiquitin ligases to activated receptors

To test whether ligand activation of receptors leads to aArr-Nedd4 family E3 binding, we used the same cell model described above: stable 293T-b2AR cells transiently transfected with ARRDC3- and ARRDC4-mCherry, respectively, and NEDD4-GFP. In the absence of receptor activation, NEDD4 expression was both diffuse and punctate, and was present in the cytoplasm and moderately enriched at the plasma membrane; and there was very minor colocalization of aArr and NEDD4 (Fig. 4B and data not shown). At 30 m post ligand stimulation, there was significantly increased colocalization of aArr and NEDD4 in a subset of cytoplasmic vesicles. Based on other evidence presented in this work (Fig. 2F), the latter seem likely to be endosomes. To biochemically test for such aArr-Nedd4 family E3 interactions, we transiently co-transfected 293T cells with HA-tagged b2AR or V2R, plus Flag-tagged ARRDC3 or 4, respectively. We then performed IP of aArr (Flag) and western blotting of select endogenous Nedd4 family E3s in unstimulated and 5 m post ligand-stimulated cells. This revealed robust interactions between aArrs and E3s (Figs. 4C–F). PY motif mutagenesis revealed that aArr-E3 interactions were PY-dependent. Ligand-stimulation effects at this timepoint were consistent with aArr time course studies (Fig. 3): there was decreased coIP of both aArrs and ITCH and WWP2, and of ARRDC4 and NEDD4L; on the other hand, there was induced coIP of ARRDC3 and NEDD4L. Notably, aArr levels were greatly increased in PY mutants and ligand activation resulted in significantly reduced levels of aArr. The findings are consistent with the possibility that overexpressed aArrs constitutively activate Nedd4 E3s. Our observations also suggest that ligand activation results in increased ubiquitination and degradation of aArrs and E3s.

**Figure 5:**
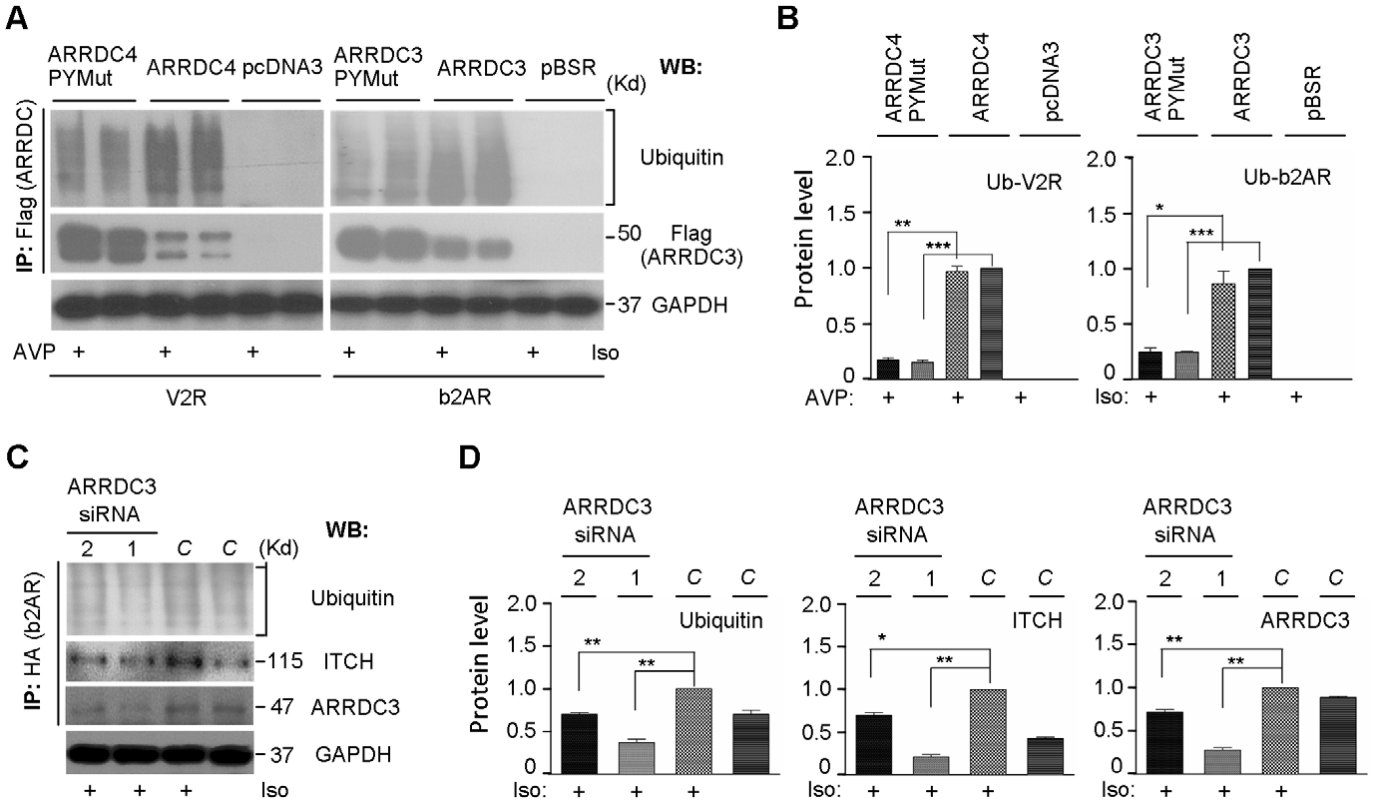
Biology of alpha arrestins. (*A*) Ubiquitination effect of alpha arrestins on GPCR. Empty vector, pBSR-ARRDC3-Flag, or pBSR-ARRDC3-Flag PY motif mutant construct was cotransfected with pcDNA3-HA-b2AR-V5. After 24 h incubation, cells were serum-starved for 2 h and treated or not with 1 uM Iso for 30 m. The transfected cells were lysed, and lysates were immunoprecipitated (IP) and analyzed by western blot (WB). The same experiment was performed with ARRDC4 and V2R (1 uM AVP treatment for 30 m). (*B*) The mean values (+/− S.D.) of three independent experiments are included in the histogram (p value levels and lines used as in Fig. 2). (*C.*) Effects of siRNA-mediated ADDRC3 knockdown on b2AR ubiquitination and ITCH expression. Scrambled siRNA, and ARRDC3 siRNAs #1 and #2 were cotransfected with HA-b2AR-V5 construct. After 48 h incubation, cells were serum-starved for 2 h and treated with 1 uM Isoproterenol Iso for 30 m. The transfected cells were lysed, and the lysates were immunoprecipitated (IP) and analyzed by western blot (WB). (*D*) Histograms of mean (+/− S.D.) and p-values calculated from three different experiments by paired, two-tailed t-tests comparing ligand-stimulated cells transfected with ARRDC3 siRNA versus with control siRNA (p value levels used as in Fig. 2).

To uncover ubiquitination biology associated with the results above, we first conducted aArr IP and probed for ubiquitin by western blotting using 293T cells transiently co-transfected with ARRDC3/b2AR or ARRDC4/V2R constructs (Fig. 5A/B). As was found in the time course study (Fig. 3), aArr IP levels were increased 30 m post ligand-activation. We again saw that aArr PY mutants are expressed at far higher levels than WT. Intriguingly; we also found that the relative amount of associated ubiquitin is much greater in WT aArr than in PY mutant. Further studies are necessary to determine how much of that ubiquitin may be attached to ARRDC3/4 and what other substrates could be present, but there is evidence that aArrs are ubiqutinated (see next section). Next we conducted similar experiments, but performed IP of b2AR (HA tag) followed by western blotting of the Nedd4 family E3 ITCH, ARRDC3 and ubiquitin (Fig. 5C/D). While ITCH and ARRDC3 signal is detected at the expected mobility for each, the ubiquitin signal is a high molecular weight smear typical for ligand-stimulated b2AR. Isoproterenol stimulation induced increases of b2AR coIP with endogenous ARRDC3, ITCH and ubiquitin.

To test whether endogenous aArr plays a role in ligand-dependent receptor ubiquitination, we used RNA interference (RNAi) knockdown of ARRDC3. We transiently co-transfected 293T cells with HA-b2AR plus either a “scrambled” control siRNA (short interfering RNA) or with one of two different ARRDC3 siRNAs (to rule out off-target effects), and confirmed the effects on ARRDC3 protein levels (Fig. 5C/D). The result of ARRDC3 knockdown was a reduction of ligand-dependent b2AR ubiquitination. That effect was proportional to the level of ARRDC3 knockdown. This is consistent with ARRDC3 recruiting Nedd4 family E3s to mediate ubiquitination of activated receptors.

### Posttranslational modifications of alpha arrestins

Yeast and invertebrate animal aArrs are known to be regulated by posttranslational modifications, including phosphorylation and ubiquitination. [20], [32], [42], [43], [44] Others have reported that the mammalian aArrs TXNIP and Arrdc1 are ubiquitinated dependent on their PY motifs, and that this results in their degradation. [24], [45], [46], [47] Use of Phosphosite mining [48] revealed mass spectroscopy evidence of TXNIP ubiquitination at 16 different lysine residues (8 of those were identified in 5 or more studies) and of ARRDC4 ubiquitination from one *in vivo* proteomic survey of ubiquitination (Fig. S3A). [49] Here we showed that aArrs appear to be degraded under conditions that result in aArr-mediated Nedd4 family E3 recruitment (compare WT and PY mutants in Figs. 2B/C; 4C/D; 5A; 6D/E). These findings suggest that aArr ubiquitination is an evolutionarily conserved aspect of aArr regulation.

**Figure 6:**
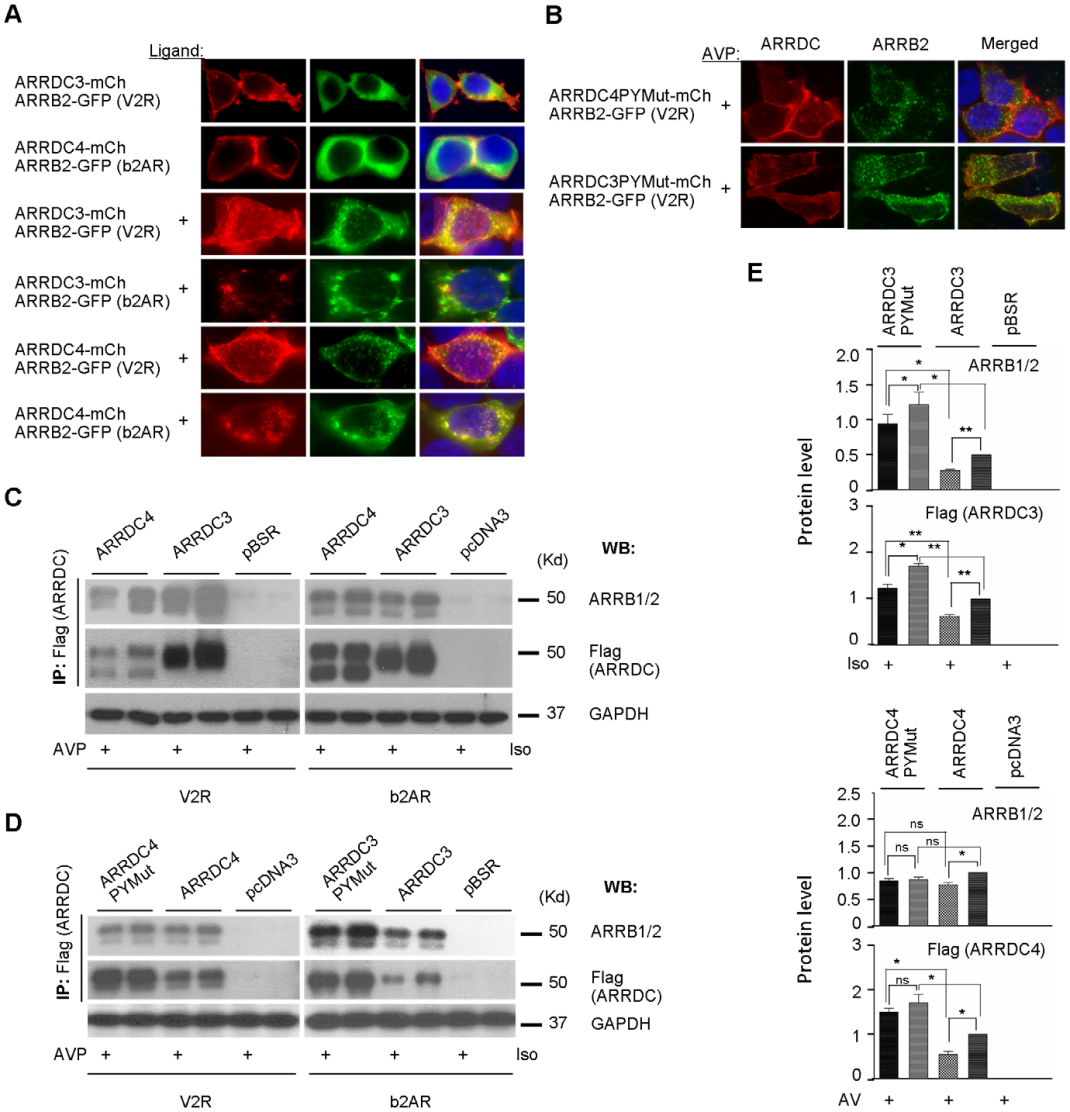
Hetero-association of alpha and beta arrestins. (*A*) Subcellular colocalization of alpha and beta arrestins in HA-b2AR and HA-V2R-V5 permanent cell lines. HA-b2AR and HA-V2R-V5 permanent cells were transiently co-transfected with either ARRDC3- or ARRDC4-mCherry construct plus pArrb2-EGFP. After 24 h, transfected cells were serum-starved for 2 h, treated or not with 1 uM ligand for 30 m, and then washed and fixed with 4% paraformaldehyde. Fluorescent confocal images were captured (aArr, *red*; ARRB2, *green*). (*B*) Subcellular colocalization of mutant alpha- and beta-arrestin in ligand treated V2R permanent cell line. HA-V2R-V5 permanent cells were transiently co-transfected with either ARRDC3- or ARRDC4-mCherry PY mutant construct plus pArrb2-EGFP. After 24 h, transfected cells were serum-starved for 2 h, treated with 1 uM AMP for 30 m, and then washed and fixed. Fluorescent confocal images were captured (aArr, *red*; ARRB2, *green*). (*C*) Co-immunoprecipitation of wild type aArrs and bArrs. HEK-293Tcells were co-transfected either with empty vector, or pBSR-ARRDC3-Flag, or pcDNA3-ARRDC4-Flag plus pcDNA3-HA-b2AR or pcDND3-HA-V2R-V5. After 24 h incubation, cells were serum-starved and treated or not with 1 uM ligand for 5 m. The cells were lysed, and lysates were immunoprecipitated and analyzed by western blot. (*D*) Co-immunoprecipitation of wild type or mutant aArrs and bArrs. HEK-293T cells were co-transfected either with empty vector, pBSR-ARRDC3-Flag, or pBSR-ARRDC3-Flag PY mutant, plus pcDNA3-HA-b2AR. Protein levels of ARRB1/2 and aArrs were detected by western blot. (*E*) The same concept as in D was done with ARRDC4 plus HA-V2R-V5. P-values were calculated by paired, two-tailed t-tests (p values and thin/bold lines used as in Fig. 2).

Yeast and invertebrate aArrs are regulated by phosphorylation and de-phosphorylation similarly to bArrs in animals. Two master regulators of metabolism in mammals – AMPK and TOR – have been shown to regulate phosphorylation of yeast aArrs directly [AMPK (Snf1) acting on aArr (Rod1)] or indirectly [TORC1 acting on Npr1, which acts on aArrs (Aly2, Art1/Ldb19)]; dephosphorylation is required for aArr ubiquitination and subsequent aArr/Nedd4 (Rsp5)-induced ubiquitination and endocytic trafficking of various carbon source and amino acid transporters. [30], [31], [32] Mining yeast protein-interaction data showed that another important kinase associated with metabolic disease in humans, GSK-3 (Mck1), also phosphorylates aArrs (Rog3, Aly2). [15] In the worm *C. elegans*, dephosphorylation of the aArr CNP-1 by calcineurin is associated with fasting and re-feeding behaviors including egg-laying, locomotion, hyperosmolarity adaptation and lysine chemotaxis. But we did not find published studies investigating aArr phosphorylation in mammals. We mined mammalian posttranslational modification data and found mass spectroscopy evidence that mammalian aArrs, including ARRDC3/4, are substrates of serine/threonine and tyrosine phosphorylation (Fig. S3A/B and data not shown). [48] Strikingly, TXNIP has eight phosphorylation sites identified in two or more studies and all are located in the Tail domain. These findings suggest mammalian aArrs are directly regulated by multiple types of posttranslational modifications similarly to yeast and invertebrate aArrs and animal bArrs.

### Alpha and beta arrestins heteroassociate

We previously noted that alpha and beta arrestins were likely to heteroassociate ([15]; discussed in [22]). Through mining of protein interaction data (BioGRID 3.1), we identified one report of an aArr-bArr interaction (high confidence yeast two hybrid interaction of *Drosophila* CG32626 and krz [50]). We also mined gene expression data to ascertain whether we could detect positive or negative correlations of aArr and bArr expression changes (using NCBI Geo, Nextbio and Oncomine analysis; not shown). The most obvious effects we found were mRNA level changes in opposite directions in a subset of metabolic and cancer studies. For example, Oncomine analysis of breast cancer data showed that ARRDC2/3/4 and TXNIP appear among the genes most downregulated between normal and cancer tissue, whereas ARRB2 appears among the genes most upregulated (Fig. S3C). [51] This is consistent with evidence that aArrs TXNIP and ARRDC3 are tumor suppressors, and that bArrs ARRB1/2 have predominantly oncogenic profiles. [25], [35], [52], [53] Our data mining observations are also consistent with opposite roles of aArrs TXNIP and ARRDC3 (pro obesogenic or diabeticogenic) compared to bArrs ARRB1 and 2 (anti). [23], [45], [54], [55] Taken together, these findings suggest that aArrs/bArrs function coordinately in the regulation of metabolism and cell growth.

To experimentally assay for coordinated aArr/bArr functions, we first performed colocalization studies using confocal microscopy of fluorescent proteins (Fig. 6A). 293T-b2AR and 293T-V2R cells were co-transfected with fluorescent protein-tagged ARRDC3 or 4 (red, mCherry), each separately, and the bArr ARRB2 (green, GFP). Unstimulated cells were essentially the same as we showed in other figures here (Figs. 1, 2A, 2F, 4B), with strong plasma membrane expression and little cytoplasmic vesicle expression of aArrs. ARRB2 in unstimulated cells had a predominantly diffuse cytoplasmic expression pattern. Looking 15 m after receptor activation with the relevant ligand, we observed dramatic increases in the numbers of fluorescent-labeled cytoplasmic vesicles and significant levels of vesicular colocalization of ARRDC3/4 and ARRB2. The same analysis of ARRDC3/4 PY mutants in 293T-V2R cells, showed that there was strong plasma membrane, but very little cytoplasmic vesicle localization of aArr PY mutants – even in ligand-stimulated cells (Fig. 6B). Strikingly, aArr PY mutants did not colocalize with ARRB2 and the latter appeared to have a normal endocytic localization pattern following ligand activation. However, we cannot rule out the possibility that endogenous alpha arrestins are somehow involved in the observed beta arrestin trafficking. These findings show that PY motifs are required for normal endocytic trafficking effects of alpha arrestins.

We also examined aArr/bArr interactions biochemically, using HEK293T cells transiently co-transfected with b2AR or V2R and ARRDC3 or 4 (Fig. 6C). IP of aArr (Flag) and western blotting for endogenous bArrs showed that aArr IP levels correlate with bArr coIP'd in both relevant ligand-stimulated and unstimulated cells. This coIP is robust in the absence of protein cross-linking, suggesting that a portion of overexpressed aArr constitutively located at the plasma membrane and cytoplasmic vesicles is associated with a minor fraction of bArr. Stimulation of cells with the relevant ligand for 5 m, showed that aArr levels and bArr coIP were generally decreased in stimulated cells. This effect is similar to that mentioned above for time course studies of liganded receptor-aArr interactions (Fig. 3). Mutagenesis of aArr PY motifs had no effect on aArr/bArr interaction (Fig. 6D/E). These various findings show that overexpressed aArr can colocalize with overexpressed bArr following ligand activation and can coIP with both endogenous bArrs.

### Arrdc3 knockout mouse

To circumvent the challenges of studying aArr biology in overexpression cell culture models, we created a gene trap *Arrdc3* knockout specifically designed to allow subsequent knockin of *Arrdc3* variants (Fig. S4). Although detailed phenotyping will take significant time, we present information here that is critically relevant to published findings. In the course of back-crossing the Arrdc3 gene trap insertion in the 129 mouse strain into the C57/BL6 strain, we identified phenotypes that were not reported in the recent publication of a similar *Arrdc3* knockout. Those two newly observed phenotypes are fully penetrant and did not change between the F4 and F7 generations (despite the fact that the genetic background is not yet pure). Patwari and colleagues recently reported metabolic studies showing that *Arrdc3* knockouts are resistant to age-induced obesity in C57/BL6 mice (using a separate insertion of the same gene trap vector into the same intron). [23] We also observed the obesity resistance and size effects, but identified two other phenotypes (Fig. S4). First, we found that homozygous *Arrdc3* KO mice are embryonic lethal when mothers are fed a normal chow diet, but not under high fat diet (Fig. S4G). Secondly, we found that all homozygous mice have very thin skin (dermis) and excessive dandruff, unlike all heterozygous or wild type mice (Fig. S4H). Notably, the skin phenotype was noted by Draheim (dissertation) in a third *Arrdc3* knockout mouse. [56] Although she was not able to genetically distinguish between homozygous and heterozygous mice, she speculated the phenotype must only be present in homozygotes. Neither of these two *Arrdc3* knockout phenotypes was mentioned by Patwari et al., but both could be related to their metabolic findings and suggest additional complexity (see Discussion). [23]

## Discussion

Although it was discovered that rodent Arrdc4 and fungal PalF are arrestins in 2004 and 2005, respectively [16], [20], it was not until 2008 that their place in the arrestin clan was characterized and aArr-specific trafficking roles were proposed. [15] That latter report noted for the first time that at least fungal and animal aArrs have evolutionarily conserved PY motifs suggestive of WW protein interactions, a hypothesis that was quickly validated in yeast. [42], [43] Recently, the first two reports of arrestin-like trafficking mechanisms of mammalian aArrs were published. [12], [25] Drahaim et al. showed that ARRDC3 induces downregulation of a specific integrin, while Nabhan et al. characterized the role of ARRDC3 in recruiting Nedd4 E3 to activated b2AR and the resulting effects of ubiquitianation and lysosomal trafficking of receptor.

Our present results (Fig. 1) confirmed prior reports of constitutively nuclear localization of TXNIP [17], [57], and constitutive localization of ARRDC3 on the plasma membrane and in cytoplasmic vesicles or endosomes [17]. ARRDC2/4 had very similar constitutive expression patterns to ARRDC3. AVP ligand activation of V2R showed altered localization of TXNIP and ARRDC1/2/3/4. In many cells, there was a clear redistribution of TXNIP upon AVP treatment, from diffuse nuclear to punctate nuclear. This is interesting because of the possibility this effect could be associated with gene regulation. It is also curious given that AVP activates V2R at the plasma membrane and this somehow induces intra-nuclear TXNIP translocation. ARRDCs 1–4 showed increased vesicular and decreased plasma membrane localization upon V2R receptor activation. This suggests that aArrs are promiscuous like bArrs. We have some evidence that ARRDC3 is more efficient than ARRDC4 at promoting ligand-dependent b2AR ubiquitination, but further studies are necessary to characterize such specificities.

Post activation time course studies of ARRDC3/b2AR or ARRDC4/V2R cotransfected cells showed a sharp increase of aArr receptor coIP at 1 m followed by levels below baseline at 5 m and then increases well above baseline that plateau at 15–30 m (Fig. 3). This and our observation of ligand-dependent colocalization of aArrs with early/late endosome markers are consistent with an aArr role in lysosomal trafficking of receptors. For the most part, the time course trends are paralleled by receptor ubiquitination and aArr levels in the same coIPs. However, at 1 m, ARRDC3/V2R showed only slightly increased coIP compared to that seen for ARRDC3/b2AR, and levels of ubiquitin and ARRDC4 that are below baseline. We speculate that this is due to accelerated kinetics of ARRDC4/V2R interactions, which could occur before our first time point of 1 m. Our time course analysis of aArrs is reminiscent of Shenoy et al.'s studies of bArr roles in b2AR signaling and lysosomal trafficking, which showed key events in the first 5 m after ligand activation: 1) increased levels of bArr, 2) ubiquitination of bArr by (RING family) E3 ligase Mdm2; 3) bArr-mediated Nedd4 E3 ligase recruitment and receptor ubiquitination, and 4) USP33 deubiquitination of bArr and its association with bArr-dependent ERK activation. [6], [11], [58] Intriguingly, coIP's of bArr-USP33 in ligand-stimulated b2AR and V2R cells show similar timing to those we see for aArr-binding and receptor ubiquitination (using the same receptors): For b2AR, both studies show a peak at the earliest timepoint of 1 m. For V2R, both studies show a decrease at 1 m, suggesting the possibility that the peak maxima occurred before 1 m. Given the high number of interactions/steric constraints, this seems more consistent with a series of transient interactions and posttranslational modifications rather than the function of a major signalosome complex.

We presented colocalization and coIP evidence that ARRDC3 and 4 can associate with Nedd4 family E3 ubiquitin ligases upon ligand activation of b2AR and V2R. Mutation of PY motifs in the aArr C-terminal Tail domains showed that they are required for coIP with Nedd4 E3s. Our yeast two hybrid analysis of isolated aArr PY motifs and Nedd4 E3 WW domains showed robust interactions (Fig. 4A). This implies that aArrs and Nedd4 family E3s interact via their respective PY motifs and WW domains. We found that overexpressed ARRDC3 and 4 can constitutively interact with and/or activate four Nedd4 family E3s (Fig. 4B–F). Throughout our coIP studies, PY mutant aArrs are expressed at far higher levels than WT. One possible explanation for this is that PY mutant aArrs have decreased turnover rates as a result of reduced interaction with and ubiquitination by Nedd4 family E3s. We conducted studies to address such protein turnover in cells co-transfected with ARRDC4 and V2R. Proteosomal inhibition with MG132 increased levels of WT (not PY mutant) ARRDC4, but the increased WT levels did not reach those of PY mutants (Fig. S2C). Inhibition of lysosomal/autophagosomal function with chloroquine actually resulted in decreased levels of both ARRDC4 and V2R. This is consistent with a partial role of proteosomal degradation in aArr turnover. IP of aArrs followed by western blotting for ubiquitin is consistent with aArrs being ubiquitinated dependent on PY motifs (Fig. 5A/B). Receptor stimulation studies show that aArr PY motifs are required for aArr endocytic trafficking (6A/B). It will be interesting to know to what extent these aspects of aArr function are related. For example, how does the plasma membrane-restricted subcellular localization of PY mutant aArrs affect their protein interactions and stability?

To determine whether ARRDC3 is involved in b2AR ubiquitination, we used siRNA knockdown of ARRDC3 in the presence of ligand stimulation (Fig. 5C/D). In control cells, isoproterenol stimulation induced increases of b2AR coIP with endogenous ARRDC3, ITCH and ubiquitin. The result of ARRDC3 knockdown was a strong reduction of ligand-dependent b2AR ubiquitination. These findings are consistent with ARRDC3 recruiting Nedd4 family E3s to mediate ubiquitination of activated receptors, as was shown in similar studies by Nabhan et al. [12] Our results with ARRDC4/V2R further suggest that these effects represent general signaling mechanisms for 7TMRs. Taken together with published studies in fungi and mammals, aArrs appear to be such endocytic trafficking adaptors for diverse cell surface proteins, including receptors, integrins and many transporters.

We previously proposed that aArrs and bArrs were likely to function coordinately. [15], [22] Here we first provided supporting evidence for this from data mining of published studies, including 1) aArr/bArr heteroassociation in the worm *C. elegans*; 2) evidence that expression of mammalian aArrs and bArrs are often coordinately regulated in opposite directions in cancer and different metabolic states; and 3) demonstration that aArrs and bArrs have opposite functional and gene expression profiles with respect to cancer [25], [35], [52], [53] and metabolism [23], [45], [54], [59], with some aArrs being tumor suppressors and obeso/diabetic-genic and bArrs being the opposite. [Our Arrdc3 knockout model is consistent with such a metabolic phenotype [23] and revealed a novel related phenotype: embryonic lethality on a normal chow diet, but not in a high fat diet.] Consistent with that body of published data, our biochemical studies show that aArrs have effects in the first 1–5 m following 7TMR signaling (Fig. 3), a time when bArrs also have multiple roles. ARRDC3 and 4 colocalize with bArr ARRB2 upon appropriate ligand activation of b2AR or V2R-transfected cells (Fig. 6A). PY mutagenesis resulted in predominant retention of ARRDC3 and 4 at the plasma membrane of activated cells, but this did not prevent plasma membrane translocation or endocytic trafficking of ARRB2 (Fig. 6B). That could suggest heteroassociation of internalized aArrs/bArrs or aArr PY motif-mediated recruitment of WW proteins are not necessary for bArr endocytic trafficking. However, it is worth noting that aArr PY mutants may not have full dominant-negative effects and endogenous aArrs may rescue transfected aArr PY mutants in our cell models. Furthermore, aArr/bArr interactions could affect endocytic trafficking kinetics, subcellular routing or destination, or they could be necessary for other biological roles that are not assayed here.

We tested for aArr/bArr coIP in cells co-transfected with ARRDC3 or 4 and b2AR or V2R, respectively. CoIP analysis in unstimulated cells showed robust interaction of aArrs and endogenous ARRB1 and 2 (Fig. 6C). Given that the majority of bArr ARRB2 in unstimulated cells did not colocalize with aArr (with relatively minor colocalization adjacent to the plasma membrane), the coIP of aArr and endogenous bArrs in unstimulated cells was at first somewhat surprising. However, this suggests the possibility that a significant portion of overexpressed aArrs, which are predominantly located at the plasma membrane in unstimulated cells, is associated with bArrs. While both ARRDC3/4 show ARRB2 colocalization upon activation of either b2AR or V2R, it is important to consider that these models have overexpression of arrestins and receptors. Studying dynamic and transient interactions in their natural state is technically very challenging. Moreover, the study of ARRB1/2 heteroassociation has been somewhat controversial and there is no consensus on, say, stoichiometry, relative orientation of binding partners, or biological effects. [60] Understanding this biochemistry and biology will likely require study of *in vivo* models expressing natural levels of wild type and mutant proteins.

### Conclusions

**Figure 7:**
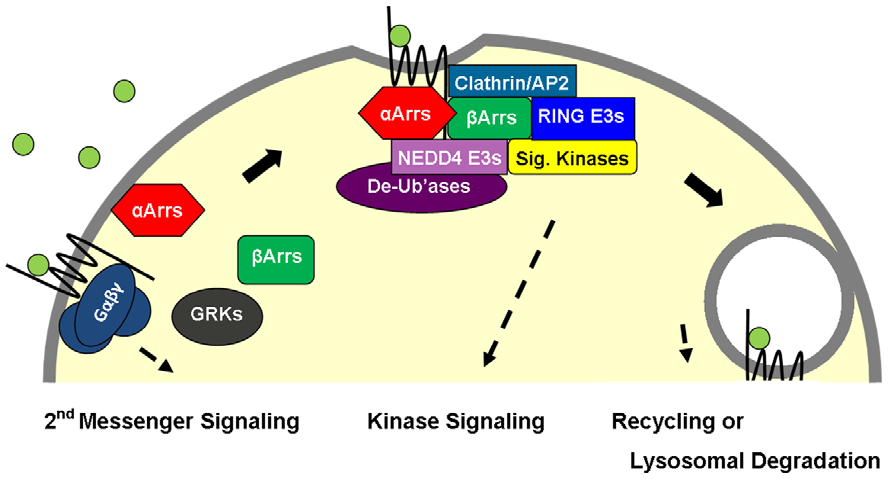
Model of integrated alpha and beta arrestin roles in membrane protein transport in mammals. In the case of 7TMRs, receptor activation results in heterotrimeric G protein signaling through second messenger systems. This is instantly followed by receptor phosphorylation by GRK, which results in beta arrestin binding (arresting further G protein binding/signaling). Our findings suggest that alpha and beta arrestins heteroassociate and have coordinated functions. Among the biological functions that could be regulated by integrated alpha/beta arrestin functions are endocytic trafficking of cell surface cargoes (recycling or lysosomal degradation), kinase signaling (e.g., metabolism, cell growth, survival/apoptosis) and cytoskeletal dynamics. Alpha and beta arrestins could have a yin yang relationship with both antagonistic (e.g., inactivating and activating, respectively) and cooperative properties depending on context. We envision that arrestins are dynamic adaptors that can integrate internal and external information to mediate programmed ends (i.e., programs created through evolutionary processes). This is largely achieved through reversible posttranslational modifications, such as phosphorylation (not shown) and ubiqutination, and their effects on protein-protein interactions.

This and previous studies paint a picture of fungal and mammalian alpha arrestins as adaptors that link activated cell surface proteins to downstream WW proteins, including Nedd4 family E3 ligases (Fig. 7). [61] We also showed evidence that alpha and beta arrestins hetero-associate, and that alphas – like betas are known to – have major biochemical roles immediately following 7TMR stimulation. It is interesting that in both yeasts and mammals, aArrs recruit Nedd4 family E3s and thus induce ligand-dependent ubiquitination of cell surface proteins. In yeasts – which predate the emergence of bArrs – that ubiquitination is necessary for both endocytosis and vacuolar (fungal lysosomal-equivalent) trafficking. However, in mammals (possibly in all animals), those functions are split between two sibling arrestin subfamilies. bArrs recruit clathrin to induce receptor endocytosis and aArrs recruit E3s to mediate receptor ubiquitination and lysosomal trafficking. While both these aArr and bArr functions would appear to promote membrane-protein downregulation, the two arrestin subfamilies have opposite effects in several physiological contexts, including in metabolism and cancer. At least some aArrs appear to be anti-cell growth/survival and obeso-/diabetico-genic, and both bArrs have the opposite profile. This hints that aArrs and bArrs have a complex yin yang relationship with both cooperative and antagonistic properties. Presumably this would relate to interactions with additional downstream proteins, such as MAGI proteins and other WW domain proteins (for aArrs) and signaling kinases (for bArrs). All these roles are likely to be dynamically regulated by aArr posttranslational modifications similarly to bArrs (as indicated by our data mining results; Fig. S3A/B). That is most strikingly suggested by TXNIP, which has eight phosphorylation sites identified in two or more proteomic studies and all are located in the Tail domain. Taken together, these findings lead us to speculate that integrated alpha/beta arrestin functions play a key role in tuning how cell surface signals are received and acted upon.

## Materials and Methods

### Ethics statement

This study was carried out in strict accordance with the recommendations in the Guide for the Care and Use of Laboratory Animals of the National Institutes of Health. The protocol was approved by the Institutional Animal Care and Use Committee of The Research Institute at Nationwide Children's Hospital (Protocol Number: AR10-00031). See Text S1, Figure S4

### Plasmids and constructs

All constructs are of human genes and were sequence verified. The C-terminal mCherry and Flag-tagged ARRDC3 (WT and PY mutant) were cloned in the pEF vector; the other aArrs were cloned in pcDNA3.1 (Invitrogen). pBSR-ARRDC3-Flag and pBSR-EGFP-ARRDC3 were generous gifts from Dr. Shinichi Oka, and pARRB2-GFP and pcDNA1.1-HA-b2AR from Dr. Laura Bohn. HA-b2AR-V5 and HA-V2R-V5 were PCR amplified to engineer the two tag fusions and to clone in pcDNA3.1. ARRDC3 and ARRDC4 clones were mutated from PPxY to PAxA (Stratagene Site-Directed Mutagenesis Kit). The mutated pEF-ARRDC3-mCherry, pEF-ARRDC4-mCherry, pBSR-ARRDC3-Flag, and pBSR-ARRDC4-Flag were used for colocalization and pull-down experiments.

### Immunological materials

Manufacturer (catalog no.): Sigma: HA-tag (2367), Clathrin (4796), ubiquitin (3933), NEDD4 (3607), NEDD4L (4013), Flag (2368), ARRB1/2 (4674), Flag M2 (F1804), anti-FLAG M2 magnetic beads (M8823), ITCH (SAB4200036), WWP2 (338–350), EZview Red Anti-HA affinity gel (E6779). Cell Signaling: GAPDH (2118), anti-mouse IgG, AP-linked (7050), anti-rabbit IgG, AP-linked (7054). Santa Cruz: c-Myc (sc-40), Hrs (sc-30221). Abcam: ARRDC3 (ab64817), ARRDC4 (ab74265). Millipore: Goat anti-rabbit IgG, HRP-conjugated (12–348); Goat anti-mouse IgG, HRP-conjugated (12–349). Thermo: Protein A/G magnetic beads (88803).

### Cell culture and transfection

HEK-293T (human embryonic kidney cells, HEK-293, constitutively expressing the SV40 large T antigen) and HeLa (human cervical cancer) cell line cells were procured from the Viral Vector Core, NCHRI. Cells were maintained in Eagle's MEM, and supplemented with 10% fetal bovine serum plus penicillin (100 units/ml) and streptomycin (100 pg/ml). Stably transfected HEK-293T cell lines were prepared and maintained in the same medium, supplemented with 300 ug/ML Hygromycin. Cells were transiently transfected with FuGENE 6 reagent (Roche).

### Yeast two-hybrid assay

Yeast two-hybrid analysis was performed using the ProQuest Two-Hybrid System (Invitrogen) according to the manufacturer's instructions. The baits were generated by PCR of ARRDC1 and ARRDC3 C-terminal tail domains, cloned in pCR8-GW-TOPO, and shuttled through the Gateway system into pDEST32 (which contains Gal4 DNA binding domain). For prey, PCR fragments of full WW domain regions (containing all 1–4 copies of WW domains) from WWP2, ITCH, SAV1, PLEKHA5, NEDD4L, and MAGI2 were Gateway-cloned into pDEST22 (which contains Gal4 Activation domain). 0.5 ug of each corresponding bait and prey plasmid were transformed into competent yeast strain MaV203. pEXP32-Krev1 plus pEXP22-RalGDS-wt was included as strong positive control, pEXP32-Krev1 plus pEXP22-RALGDS-m1 as weak positive control, and pEXP32-Krev1 plus pEXP22-RALGDS-m2 and empty bait vector pDEST32 plus empty prey vector pDEST22 as negative controls. Positive clones growing on SD/-Trp/-Leu/-His/-Ura plates were selected and the activity of lacZ reporter gene was examined by colony-lift filter assay.

### *In vivo* colocalization and confocal microscopy

HEK293T cells, or HA-b2AR, or HA-V2R-V5 permanent cell lines were seeded in L-poly-Lysine-treated 4-well chambered slides for two to three days. 24 h after transient transfection, cells were stimulated with corresponding ligand. Cells were subsequently washed and fixed with 4% paraformaldehyde for 10 m, then blocked and permeabilized with 5% bovine serum albumin (BSA), and Triton X-100 for 30 m. Anti-HA or other primary antibody was used to detect the fusion protein when non-fluorescent tagged-constructs were used, followed by fluorochrome-conjugated secondary antibodies against the corresponding species. We used the Zeiss 510 META and AxioVision 4.7 to obtain confocal images.

### Immunoprecipitation and western blotting

After transfection of the constructs, cells were serum-starved for 2 h and administered the corresponding ligand. Cells were then solubilized in RIPA buffer with proteinase and phosphatase inhibitors: 20 mM Tris-HCl (pH 7.5), 150 mM NaCl, 1 mM Na2EDTA, 1 mM EGTA, 1% NP-40, 1% sodium deoxycholate, 2.5 mM sodium pyrophosphate, 1 mM b-glycerophosphate, 1 mM Na_3_VO_4_, 1 µg/ml leupeptin. The deubiquitinase inhibitor N-ethylmaleimide (NEM; 10 mM) was also included. Lysates were first immunoprecipitated with EZview Red Anti-HA affinity gel, or ANTI-Flag M2 Magnetic beads (both from Sigma), or in some cases Protein A/G magnetic beads (Thermo). Immune complexes were eluted with synthetic peptides, and boiled in SDS sample buffer. The soluble proteins are resolved on a SDS-PAGE and transferred to a PVDF membrane. Chemiluminescent detection was performed using Immobilon western substrate (Millipore). All experiments were conducted in the absence of crosslinking agents.

### Quantification and statistic analysis

Protein bands were quantified by densitometry and analyzed with ImageJ. [62] Plotting and statistical analysis were done from three independent experiments including the mean (+/− S.D.) and p-values calculated by paired, two-tailed t-tests (Prism5, GraphPad Software, Inc.). Significance is indicated as ***, p<0.001; **, p<0.01; *p<0.05.

## Supporting Information

Figure S1 **Evolutionary conservation of alpha arrestin PY motifs.** Multiple sequence alignment of alpha arrestin Tail domains from vertebrate ARRDC2, 3, 4, and TXNIP show evolutionary conservation of PY motifs. BLAST analysis was used to identify arrestin orthologues from representative species (all but ARRDC4 have orthologues in human, chick, frog and fish; ARRDC4 has no fish orthologue; Ref.1). Alignment was conducted with Clustal W and the consensus calculation and display were generated in the MView multiple alignment viewer and adapted to highlight conservation of PY (aka (L/P)PxY) motifs in black highlighting and other conserved residues in yellow. The full length C-terminal Tail domain shown is based on published domain mapping.1 Accession numbers follow: Arrdc4_chick Gallus_XP_413881.2; ARRDC4_human, Homo_NP_899232.2; Arrdc4_frog, Xenopus_s_NP_001107732.1; Arrdc2_fish, Danio_AAH68345.1; Arrdc2_frog, Xenopus-l_AAH71094.1; Arrdc2_chick, Gallus_XP_001233360.1; ARRDC2-1_human, Homo_NP_056498; Arrdc3_chick, Gallus_XP_424699.2; ARRDC3_human, Homo_ref_NP_065852.1; Arrdc3_frog, Xenopus_l_assmbl-NM_001096667.1; Arrdc3_fish, Danio_NP_001073498.1; TXNIP_human, Homo_NP_006463.2; Txnip_chick, Gallus_transl-BX933080.1; Txnip_frog, Xenopus_l_AAH77193.1; Txnip_fish, Danio-1_NP_956381.1. Reference: 1. Alvarez, C.E. On the origins of arrestin and rhodopsin. BMC Evol Biol 8, 222 (2008).
(TIFF)

Figure S2 **Specificity of coIP western blotting for aArrs and receptors, and proteosomal/lysosomal inhibition studies of aArr stability.** (*A*) aArrs and 7TMRs are specifically detected by coIP/western blotting. Here we rule out the possibility that those 50 kD western bands are the result of contamination with IgG from the IP step. CoIP of aArr ARRDC3 with b2AR is shown: HEK-293T cells were transiently cotransfected with HA-b2AR-V5 plus either empty vector, pBSR-ARRDC3-GFP, or pBSR-ARRDC3 PY motif mutant construct respectively. After 24 h incubation, cells were serum-starved for 2 h and treated or not with 1 uM Iso for 30 m. The cells were lysed, and lysates were immunoprecipitated (IP) and analyzed by western blot (WB). See “No receptor” controls have no 50 kD bands due to contamination with Ig heavy band. (*B*) Ligand-activation enhances receptor-aArr interaction. aArrs and 7TMRs are specifically detected by coIP/western blotting. Here we again rule out the possibility that the 50 kD western bands are the result of contamination with IgG from the IP step (see “No receptor” controls). CoIP of aArr ARRDC4 with V2R is shown: HEK-293T cells were transiently cotransfected with HA-V2R plus either empty vector, pcDNA3-ARRDC4-GFP, or pcDNA3-ARRDC4 PY motif mutant construct respectively. After 24 h incubation, cells were serum-starved for 2 h and treated or not with 1 uM AVP for 30 m. (*C*) Effects of proteosomal and lysosomal/autophagosomal inhibitors on aArr levels. HeLa cells were chosen as they are more viable under these treatments than HEK293T cells. Cells were transiently cotransfected with HA-V2R-V5 plus either empty vector, pcDNA3-ARRDC4-Flag, or pcDNA3-ARRDC4-Flag PYmut construct. After 24 h incubation, cells were serum-starved and, at the same time, treated or not with 1 uM AVP plus either 10 uM MG-132 or 100 uM chloroquine for 3 h. The cells were lysed, and lysates were analyzed by western blotting (WB). (*D*) Time course analysis showing specificity of aArr and 7TMR coIP/western. HEK-293T cells were transiently cotransfected with HA-b2AR-V5 plus pBSR-ARRDC3-Flag. Receptor only and ARRDC3 only were also included as controls. After 24 h incubation, cells were serum-starved and treated with 1 uM Iso for 1, 5, 15, 30, and 60 m. The cells were lysed, ARRDC3 was IP'd, and ubiquitin and ARRDC3 were detected by western blotting.
(TIF)

Figure S3 **Post-translational modifications of aArrs and gene expression analysis of aArrs and bArrs in cancer.** (A, B) Use of PhosphositePlus to mine posttranslational modifications of ARRDC3/4 reveals residues that are ubiquitinated (-u; protein location mapped in schematic and sequence context shown below) and phosphorylated (-p).1 MS No. preceding the modification and sequence corresponds to the number of separate studies that identified the modification using discovery mode mass spectrometry. (C) Oncomine analysis of publicly available breast cancer gene expression data. We identified all studies/datasets where ARRDC2-4, TXNIP and ARRB2 are differentially expressed between matched cancer and non-cancer tissues, and where the relative expression change places that gene in the top 1 or 5% of the most changed genes (both decreased and increased expression). These findings show a strong trend of reduced expression of aArrs and increased expression of bArrs in breast cancer. References: 1) Hornbeck, P. V. et al. PhosphoSitePlus: a comprehensive resource for investigating the structure and function of experimentally determined post-translational modifications in man and mouse. Nucleic Acids Res 40, D261–270, doi:10.1093/nar/gkr1122 (2012); 2) Rhodes, D. R. et al. Oncomine 3.0: genes, pathways, and networks in a collection of 18,000 cancer gene expression profiles. Neoplasia 9, 166–180 (2007).
(TIF)

Figure S4 **Arrdc3 knockout mouse.** (A) We acquired Bay Genomics ES cell line CSE513, which is a gene trap insertion of the pGT0lxf gene trap vector (see Methods) and conducted a blastocyst injection (parental ES 129 line; blastocyst C57BL/6). (B) Genotyping with assay to detect gene trap vector (musArrdc3ex1L -Geo5prR, Methods) showed germ line transmission from blastocyst injection chimeras was successful. The positive control is vector DNA and the negative is WT mouse DNA. (C) Sequencing of the PCR product from the gene trap to Arrdc3 exons 1 and 2 (D) established the location of the insertion in intron 1, as shown here using the UCSC Genome browser. (E) Based on the location, we optimized a PCR Genotyping assay (En2inL-Geo5prR, Methods) of the insertion site (left) and a mouse sex control assay for PCR quality (right; Mouse_XY, Methods). A positive band demonstrates presence of at least one allele containing the vector. (F) To determine homozygosity status, we designed a PCR assay to compliment En2inL-Geo5prR (Arr-GT-in1-L/Arr-GT-in1-R; Methods). A positive band demonstrates presence of at least one WT allele. (G) Arrdc3 knockout mice were placed on a normal mouse diet containing 18% protein, 6% fat (Harlan Teklad 2018) and heterozygous crosses were performed [(+/−)×(+/−)]. The 4 resultant litters were considerably smaller in total litter size than expected and none of the offspring were homozygous for the vector. Subsequently, the breeders were placed on a high fat diet containing 23% protein, 21% fat (PicoLab Mouse Diet 20, PMI Nutrition International LLC). Shown here is a comparison of the two groups (blue, 4 litters on the normal diet; red, 5 litters on the high-fat diet) for genotyped offspring that were homozygous WT, homozygous-α (vector), and heterozygous (all were generated between the F4–F7 generation to back-cross the gene trap insertion in strain 129 into strain C57/BL6). Current sample size comparisons do not have sufficient power to parse out statistical significance within genotyped groups (p = 0.164). (H) Genotypes of littermates from crosses of heterozygotes are always apparent by their size. Note that homozygous mutants are smaller, heterozygotes are intermediate and homozygous wild type are larger. Inset shows closeup of the coat of homozygous Arrdc3 mutant. The light colored speckling is due to dandruff (flakes of dead skin), which is associated with very thin skin in homozygous mutants only. Animals shown are from the F5 generation; the Arrdc3 WT mouse shows increased contribution to coat color from the 129 strain relative to its littermates.
(TIF)

Text S1 **Materials and Methods: Arrdc3 knockout mouse.** Design and genotyping analysis of *Arrdc3* gene trap knockout mouse.
(DOCX)
