# Supplementary figures and images for: Mammalian Alpha Arrestins Link Activated Seven Transmembrane Receptors to Nedd4 Family E3 Ubiquitin Ligases and Interact with Beta Arrestins
Source: PLoS One. 2012 Dec 7;7(12):e50557. doi: 10.1371/journal.pone.0050557 (PMC3517545; doi:10.1371/journal.pone.0050557)

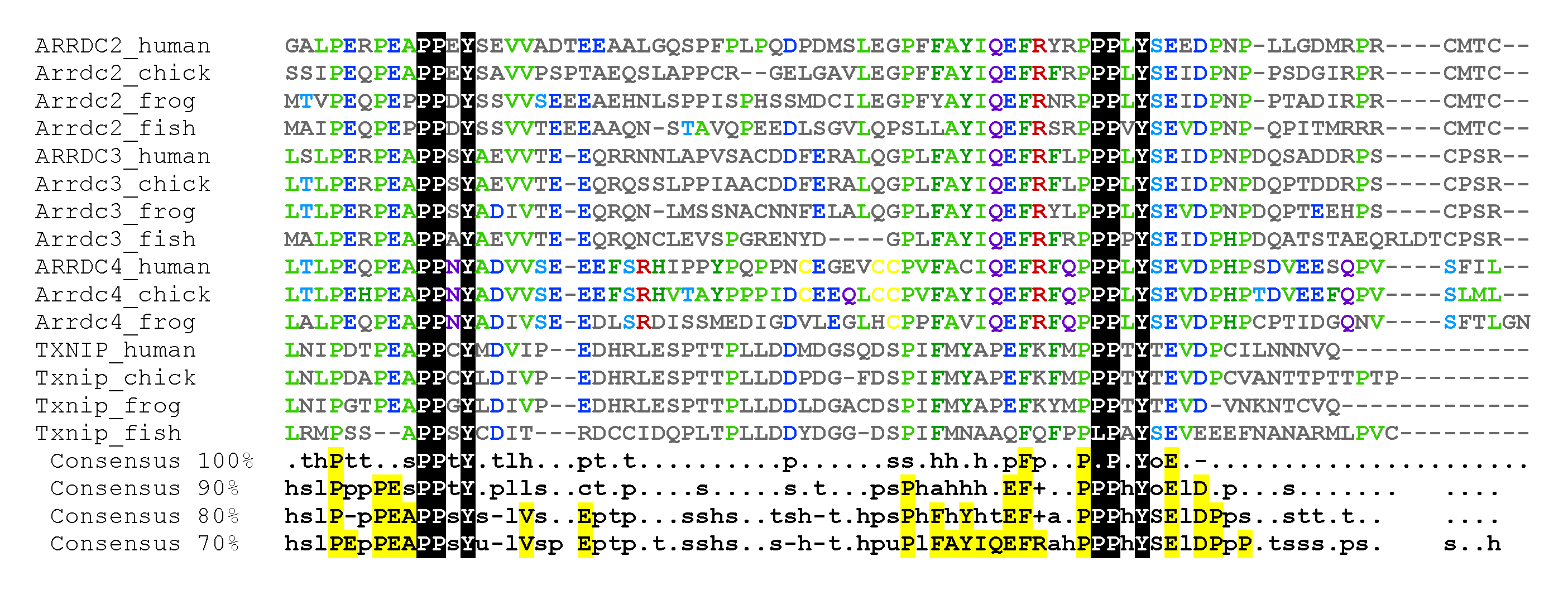

Supplement: Figure S1 — Evolutionary conservation of alpha arrestin PY motifs. Multiple sequence alignment of alpha arrestin Tail domains from vertebrate ARRDC2, 3, 4, and TXNIP show evolutionary conservation of PY motifs. BLAST analysis was used to identify arrestin orthologues from representative species (all but ARRDC4 have orthologues in human, chick, frog and fish; ARRDC4 has no fish orthologue; Ref.1). Alignment was conducted with Clustal W and the consensus calculation and display were generated in the MView multiple alignment viewer and adapted to highlight conservation of PY (aka (L/P)PxY) motifs in black highlighting and other conserved residues in yellow. The full length C-terminal Tail domain shown is based on published domain mapping.1 Accession numbers follow: Arrdc4_chick Gallus_XP_413881.2; ARRDC4_human, Homo_NP_899232.2; Arrdc4_frog, Xenopus_s_NP_001107732.1; Arrdc2_fish, Danio_AAH68345.1; Arrdc2_frog, Xenopus-l_AAH71094.1; Arrdc2_chick, Gallus_XP_001233360.1; ARRDC2-1_human, Homo_NP_056498; Arrdc3_chick, Gallus_XP_424699.2; ARRDC3_human, Homo_ref_NP_065852.1; Arrdc3_frog, Xenopus_l_assmbl-NM_001096667.1; Arrdc3_fish, Danio_NP_001073498.1; TXNIP_human, Homo_NP_006463.2; Txnip_chick, Gallus_transl-BX933080.1; Txnip_frog, Xenopus_l_AAH77193.1; Txnip_fish, Danio-1_NP_956381.1. Reference: 1. Alvarez, C.E. On the origins of arrestin and rhodopsin. BMC Evol Biol 8, 222 (2008). (TIFF) [file pone.0050557.s001.tiff]

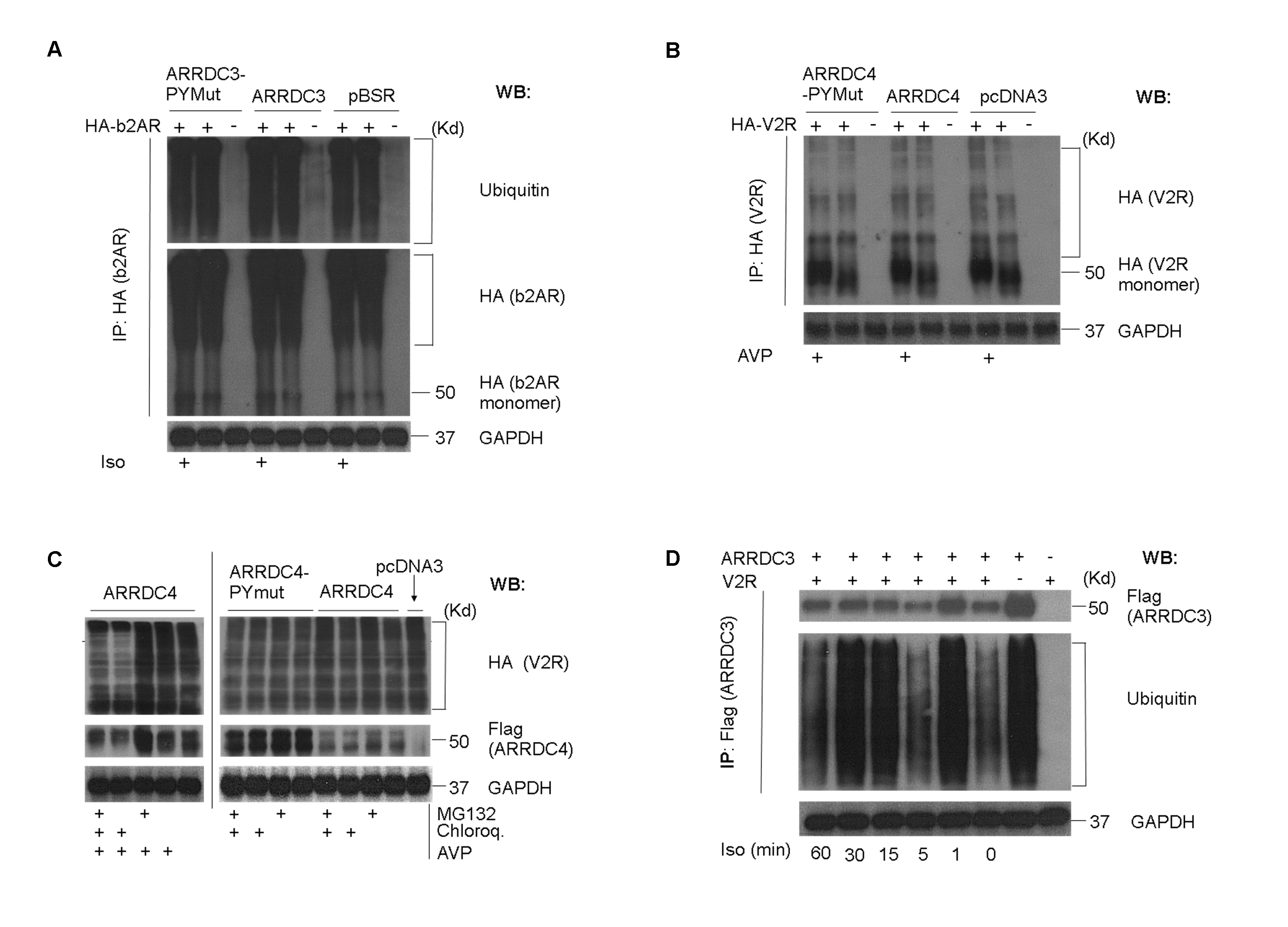

Supplement: Figure S2 — Specificity of coIP western blotting for aArrs and receptors, and proteosomal/lysosomal inhibition studies of aArr stability. (A) aArrs and 7TMRs are specifically detected by coIP/western blotting. Here we rule out the possibility that those 50 kD western bands are the result of contamination with IgG from the IP step. CoIP of aArr ARRDC3 with b2AR is shown: HEK-293T cells were transiently cotransfected with HA-b2AR-V5 plus either empty vector, pBSR-ARRDC3-GFP, or pBSR-ARRDC3 PY motif mutant construct respectively. After 24 h incubation, cells were serum-starved for 2 h and treated or not with 1 uM Iso for 30 m. The cells were lysed, and lysates were immunoprecipitated (IP) and analyzed by western blot (WB). See “No receptor” controls have no 50 kD bands due to contamination with Ig heavy band. (B) Ligand-activation enhances receptor-aArr interaction. aArrs and 7TMRs are specifically detected by coIP/western blotting. Here we again rule out the possibility that the 50 kD western bands are the result of contamination with IgG from the IP step (see “No receptor” controls). CoIP of aArr ARRDC4 with V2R is shown: HEK-293T cells were transiently cotransfected with HA-V2R plus either empty vector, pcDNA3-ARRDC4-GFP, or pcDNA3-ARRDC4 PY motif mutant construct respectively. After 24 h incubation, cells were serum-starved for 2 h and treated or not with 1 uM AVP for 30 m. (C) Effects of proteosomal and lysosomal/autophagosomal inhibitors on aArr levels. HeLa cells were chosen as they are more viable under these treatments than HEK293T cells. Cells were transiently cotransfected with HA-V2R-V5 plus either empty vector, pcDNA3-ARRDC4-Flag, or pcDNA3-ARRDC4-Flag PYmut construct. After 24 h incubation, cells were serum-starved and, at the same time, treated or not with 1 uM AVP plus either 10 uM MG-132 or 100 uM chloroquine for 3 h. The cells were lysed, and lysates were analyzed by western blotting (WB). (D) Time course analysis showing specificity of aArr and 7TMR coIP/western [file pone.0050557.s002.tif]

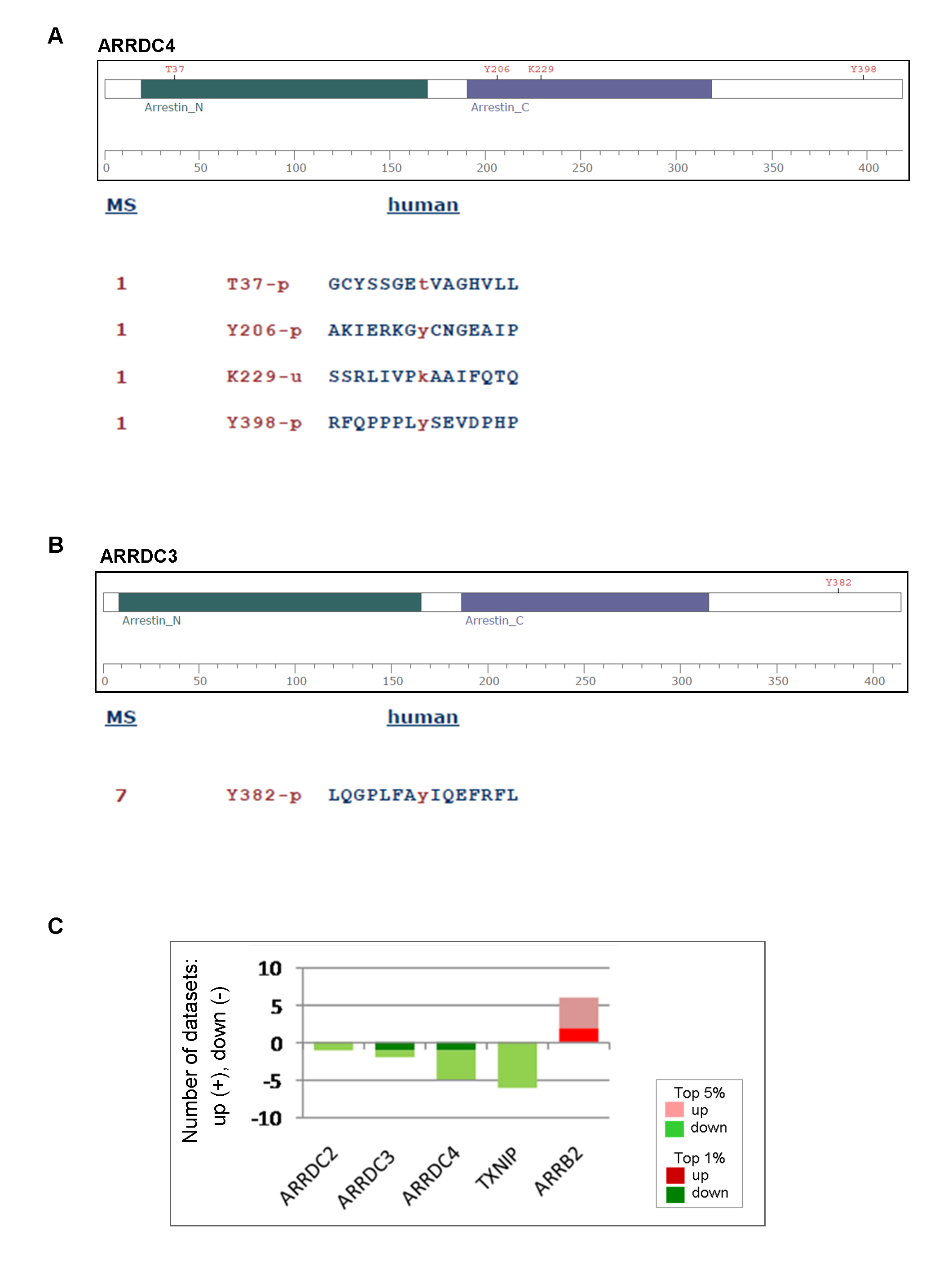

Supplement: Figure S3 — Post-translational modifications of aArrs and gene expression analysis of aArrs and bArrs in cancer. (A, B) Use of PhosphositePlus to mine posttranslational modifications of ARRDC3/4 reveals residues that are ubiquitinated (-u; protein location mapped in schematic and sequence context shown below) and phosphorylated (-p).1 MS No. preceding the modification and sequence corresponds to the number of separate studies that identified the modification using discovery mode mass spectrometry. (C) Oncomine analysis of publicly available breast cancer gene expression data. We identified all studies/datasets where ARRDC2-4, TXNIP and ARRB2 are differentially expressed between matched cancer and non-cancer tissues, and where the relative expression change places that gene in the top 1 or 5% of the most changed genes (both decreased and increased expression). These findings show a strong trend of reduced expression of aArrs and increased expression of bArrs in breast cancer. References: 1) Hornbeck, P. V. et al. PhosphoSitePlus: a comprehensive resource for investigating the structure and function of experimentally determined post-translational modifications in man and mouse. Nucleic Acids Res 40, D261–270, doi:10.1093/nar/gkr1122 (2012); 2) Rhodes, D. R. et al. Oncomine 3.0: genes, pathways, and networks in a collection of 18,000 cancer gene expression profiles. Neoplasia 9, 166–180 (2007). (TIF) [file pone.0050557.s003.tif]

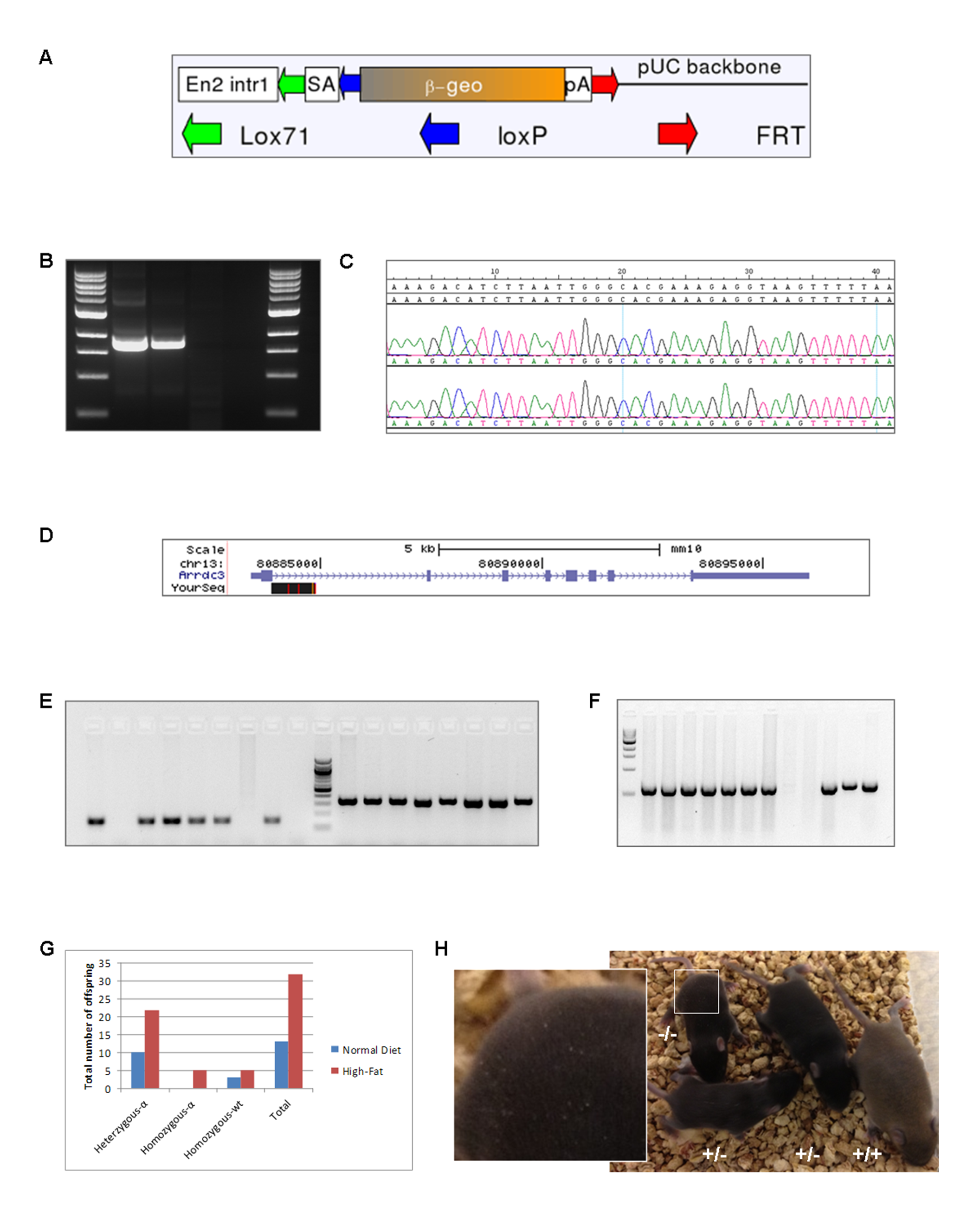

Supplement: Figure S4 — Arrdc3 knockout mouse. (A) We acquired Bay Genomics ES cell line CSE513, which is a gene trap insertion of the pGT0lxf gene trap vector (see Methods) and conducted a blastocyst injection (parental ES 129 line; blastocyst C57BL/6). (B) Genotyping with assay to detect gene trap vector (musArrdc3ex1L -Geo5prR, Methods) showed germ line transmission from blastocyst injection chimeras was successful. The positive control is vector DNA and the negative is WT mouse DNA. (C) Sequencing of the PCR product from the gene trap to Arrdc3 exons 1 and 2 (D) established the location of the insertion in intron 1, as shown here using the UCSC Genome browser. (E) Based on the location, we optimized a PCR Genotyping assay (En2inL-Geo5prR, Methods) of the insertion site (left) and a mouse sex control assay for PCR quality (right; Mouse_XY, Methods). A positive band demonstrates presence of at least one allele containing the vector. (F) To determine homozygosity status, we designed a PCR assay to compliment En2inL-Geo5prR (Arr-GT-in1-L/Arr-GT-in1-R; Methods). A positive band demonstrates presence of at least one WT allele. (G) Arrdc3 knockout mice were placed on a normal mouse diet containing 18% protein, 6% fat (Harlan Teklad 2018) and heterozygous crosses were performed [(+/−)×(+/−)]. The 4 resultant litters were considerably smaller in total litter size than expected and none of the offspring were homozygous for the vector. Subsequently, the breeders were placed on a high fat diet containing 23% protein, 21% fat (PicoLab Mouse Diet 20, PMI Nutrition International LLC). Shown here is a comparison of the two groups (blue, 4 litters on the normal diet; red, 5 litters on the high-fat diet) for genotyped offspring that were homozygous WT, homozygous-α (vector), and heterozygous (all were generated between the F4–F7 generation to back-cross the gene trap insertion in strain 129 into strain C57/BL6). Current sample size comparisons do not have sufficient power to parse out statistical signif [file pone.0050557.s004.tif]
