# Supplementary material for: Mammalian Alpha Arrestins Link Activated Seven Transmembrane Receptors to Nedd4 Family E3 Ubiquitin Ligases and Interact with Beta Arrestins
Source: PLoS One. 2012 Dec 7;7(12):e50557. doi: 10.1371/journal.pone.0050557 (PMC3517545; doi:10.1371/journal.pone.0050557)
Supplement: Text S1 — Materials and Methods: Arrdc3 knockout mouse. Design and genotyping analysis of Arrdc3 gene trap knockout mouse. (DOCX) [file pone.0050557.s005.docx]

**Text S1. Materials and Methods: Arrdc3 knockout mouse**

*Mouse Arrdc3 gene trap knockout*

This study was carried out in strict accordance with the recommendations in the Guide for the Care and Use of Laboratory Animals of the National Institutes of Health. The protocol was approved by the Institutional Animal Care and Use Committee of The Research Institute at Nationwide Children’s Hospital (Protocol Number: AR10-00031). We acquired Bay Genomics ES cell line CSE513 (Parental ES cell 129/Ola mouse strain), which corresponds to the pGT0lxf gene trap vector inserted into the first intron of Arrdc3 (insertion site sequence-confirmed, Murine Stem Cell Laboratory, Mutant Mouse Regional Resource Center, UC Davis). Blastocyst injection (C57BL/6 mouse strain) was performed by the Transgenic and Embryonic Stem Cell Core at The Research Institute at Nationwide Children's Hospital. Three different chimeric lines were backcrossed into C57BL/6. Gene trap vector information can be found at the International Gene Trap Consortium (IGTC), the Sanger Institute Gene Trap Resource (SIGTR), http://www.sanger.ac.uk/PostGenomics/genetrap/vectors/.

*Mouse Arrdc3 gene trap genotyping*

PCR methods were carried out per the JumpStart REDTaq DNA Polyermase protocol (Sigma-Aldrich), with optimizations of each different primer pair reaction. PCR primers were custom designed using Oligo Calc Nearest Neighbor based melting temperature (TM) calculations to have TM of approximately 60C (primers listed below) [2], and then primer target specificity verified using the UCSC Genome Browser GRCm38/mm10 assembly [3]. Oligos were generated by Integrated DNA Technologies (IDT). The genotyping assays below were designed to (1) verify presence or absence of pGT0lxf gene trap vector, (2) determine homozygosity of WT or pGT0lxf gene trap vector and, (3) verify the quality of the gDNA used for PCR by running a control PCR on mouse sex. We established these genotyping methods through PCR assays and sequencing analysis using ES cell vector and negative WT mouse DNA. A band present in En2inL-Geo5prR but absent in Arr_GT_in1_L/ Arr_GT_in1_R, establishes homozygosity of pGT0lxf gene trap vector. A band absent in En2inL-Geo5prR but present in Arr-GT-in1-L/ Arr-GT-in1-R establishes WT homozygosity. A band present in both assays indicates heterzogosity.

*Primers to establish Insert Location:*

musArrdc3ex1L,GGC TCC AAT ACT GCC TAT ACA CAG

Geo5prR, AGT CCA GAT CTG CGA TAA CTT CG

*Genotyping assay primers:*

1. En2inL, GTA GCT AGA CTC CAG CAA CCA G

Geo5prR, AGT CCA GAT CTG CGA TAA CTT CG

2. Arr_GT_in1_L, ATC CTG TTT CGG ACC AGT TCT GGG

Arr_GT_in1_R, ACC GTG AGA TCG TGG TGC AAT G

3. mouse_XY_F, TGA AGC TTT TGG CTT TGA G

mouse_XY_R, CCG CTG CCA AAT TCT TTG G

*Tissue*

gDNA was isolated from toe clippings obtained from pups <14 days old following established protocols [4,5]. We performed a modified Puregene (Gentra) Genomic DNA purification Kit protocol, with an additional 55C water bath overnight incubation with proteinase K.

**References:**

1. Kibbe WA (2007) OligoCalc: an online oligonucleotide properties calculator. Nucleic Acids Res 35: W43-46.

2. Karolchik D, Baertsch R, Diekhans M, Furey TS, Hinrichs A, et al. (2003) The UCSC Genome Browser Database. Nucleic Acids Res 31: 51-54.

3. Lawson PT, American Association for Laboratory Animal S (1999) Assistant laboratory animal technician : ALAT training manual. Memphis, Tenn.: American Association for Laboratory Animal Science.

4. National Research Council . Committee for the Update of the Guide for the C, Use of Laboratory A, Institute for Laboratory Animal R, National Academies P (2011) Guide for the care and use of laboratory animals. Washington, D.C.: National Academies Press.

5. Science USOo, Technology P (1985) Laboratory animal welfare; U.S. government principles for the utilization and care of vertebrate animals used in testing, research and training; notice. Federal register 50: 20864-20865.
